# Elucidating the Role of Antisolvents on the Surface Chemistry and Optoelectronic Properties of CsPbBr_*x*_I_3-x_ Perovskite Nanocrystals

**DOI:** 10.1021/jacs.2c02631

**Published:** 2022-06-27

**Authors:** Junzhi Ye, Zhenchao Li, Dominik J. Kubicki, Yunwei Zhang, Linjie Dai, Clara Otero-Martínez, Manuel A. Reus, Rakesh Arul, Kavya Reddy Dudipala, Zahra Andaji-Garmaroudi, Yi-Teng Huang, Zewei Li, Ziming Chen, Peter Müller-Buschbaum, Hin-Lap Yip, Samuel D. Stranks, Clare P. Grey, Jeremy J. Baumberg, Neil C. Greenham, Lakshminarayana Polavarapu, Akshay Rao, Robert L. Z. Hoye

**Affiliations:** † Cavendish Laboratory, University of Cambridge, JJ Thomson Ave, Cambridge CB3 0HE, United Kingdom; ‡ State Key Laboratory of Luminescent Materials and Devices, School of Materials Science and Engineering, South China University of Technology, 381 Wushan Road, Guangzhou 510640, China; § Yusuf Hamied Department of Chemistry, University of Cambridge, Lensfield Road, Cambridge CB2 1EW, United Kingdom; ∥ School of Physics, Sun Yat-sen University, 510275 Guangzhou, China; # CINBIO, Universidade de Vigo, Materials Chemistry and Physics Group, Department of Physical Chemistry, Campus Universitario As Lagoas, Marcosende, 36310 Vigo, Spain; ⊥ Lehrstuhl für Funktionelle Materialien, Physik-Department, Technische Universität München, James-Franck-Str. 1, 85748 Garching, Germany; ∇ Heinz Maier-Leibnitz Zentrum (MLZ), Technische Universität München, Lichtenbergstr. 1, 85748 Garching, Germany; ○ Department of Materials Science and Engineering, City University of Hong Kong, Tat Chee Avenue, Kowloon, Hong Kong; ◆ Department of Chemical Engineering & Biotechnology, University of Cambridge, Cambridge CB3 0AS, United Kingdom; ¶ Department of Materials, Imperial College London, Exhibition Road, London SW7 2AZ, United Kingdom

## Abstract

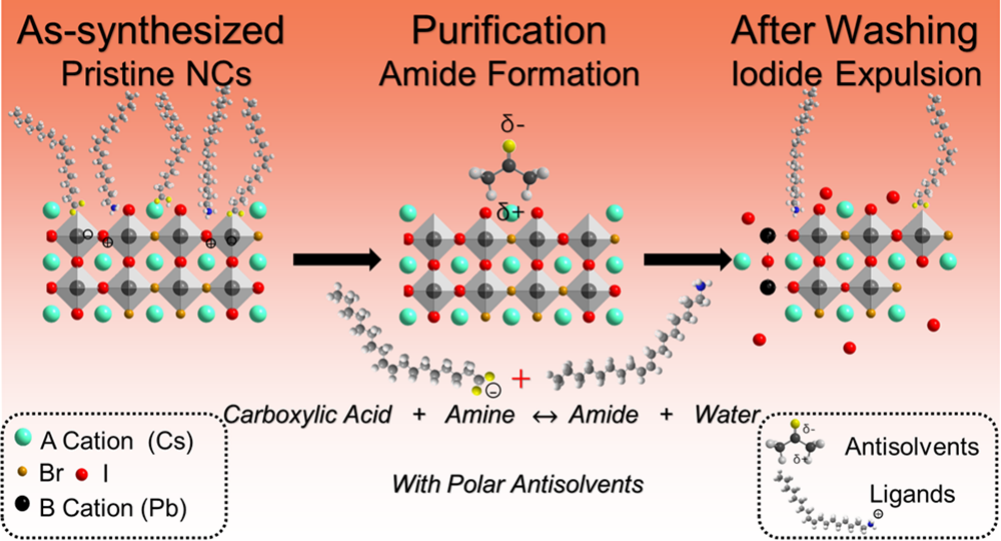

Colloidal lead-halide perovskite nanocrystals (LHP NCs) have emerged over the past decade as leading candidates for efficient next-generation optoelectronic devices, but their properties and performance critically depend on how they are purified. While antisolvents are widely used for purification, a detailed understanding of how the polarity of the antisolvent influences the surface chemistry and composition of the NCs is missing in the field. Here, we fill this knowledge gap by studying the surface chemistry of purified CsPbBr_*x*_I_3-*x*_ NCs as the model system, which in itself is considered a promising candidate for pure-red light-emitting diodes and top-cells for tandem photovoltaics. Interestingly, we find that as the polarity of the antisolvent increases (from methyl acetate to acetone to butanol), there is a blueshift in the photoluminescence (PL) peak of the NCs along with a decrease in PL quantum yield (PLQY). Through transmission electron microscopy and X-ray photoemission spectroscopy measurements, we find that these changes in PL properties arise from antisolvent-induced iodide removal, which leads to a change in halide composition and, thus, the bandgap. Using detailed nuclear magnetic resonance (NMR) and Fourier-transform infrared spectroscopy (FTIR) measurements along with density functional theory calculations, we propose that more polar antisolvents favor the detachment of the oleic acid and oleylamine ligands, which undergo amide condensation reactions, leading to the removal of iodide anions from the NC surface bound to these ligands. This work shows that careful selection of low-polarity antisolvents is a critical part of designing the synthesis of NCs to achieve high PLQYs with minimal defect-mediated phase segregation.

## Introduction

Cesium lead halide perovskite nanocrystals (NCs) have drawn significant attention due to their superior optoelectronic properties, which include their efficient luminescence and narrow emission peak that is spectrally tunable over the entire visible wavelength range.^1−8^ Since Kovalenko and co-authors established a simple hot injection synthesis method for CsPbX_3_ (X = Cl, Br, or I) NCs in 2015,^8^ these all-inorganic perovskite NCs have emerged as an appealing class of materials for ultrahigh-definition display (via light-emitting diodes or LEDs),^3,7,9−12^ lasing,^3,13^ and photovoltaic applications.^3,9,14,15^ For all of these applications, it is important to be able to fine-tune the optical bandgap, and this is commonly achieved by mixing species in the halide site, such as Br/Cl for blue-emitters or Br/I for red-emitters according to the Rec. 2020 standard.^16−23^

One of the essential steps to obtain low polydispersity NCs for LED devices is purification. This step is needed to remove impurities such as unreacted precursors or ligands, which can significantly affect film formation and device performance. The solubility of perovskites in different solvents is related to its relative polarity (which is correlated to the dielectric constant or ε_r_), in which more polar solvents have a stronger dipole moment and, therefore, a greater ability to dissociate bonded species to cause dissolution. The as-synthesized NCs are usually capped with long-chain alkyl ligands (namely oleic acid (OA) or oleylamine (OAm)) and dissolved in “good” solvents, such as toluene (ε_r_ = 2.24; relative polarity = 0.099), which are typically non-polar and have low dielectric constants. During purification, “poor” solvents (i.e., antisolvents, which are polar, with high dielectric constants), such as alcohols, are added to precipitate the NCs from “good” solvents, leaving behind impurities in the original solution (the supernatant). However, several groups have observed that the optoelectronic properties of the resulting NCs depend on the antisolvent used.^24−26^ For example, Hoshi et al. observed that the photoluminescence quantum yield (PLQY) of CsPbBr_3_ NCs decreased when washing with polar solvents, such as acetonitrile, acetone and methanol.^26^ Similarly, for mixed-halide perovskite NCs, such as CsPbBr_*x*_I_3-*x*_ NCs, selective etching was observed when the NCs were purified with acetone. That is, iodides were removed during the purification process, while bromides were retained in the NCs.^27^ As a result, the photoluminescence (PL) peak blueshifted, and the PLQY decreased after purification. These indicate that an increase in the bandgap occurred due to changes in mixed halide composition caused by a partial removal of iodide from the NC surface.^20,27−29^ However, the cause of the decrease in PLQY, removal of surface halides after washing, and relationship between the selective etching of iodides and the properties of the antisolvents used during washing remains unclear. Hints on how the surface chemistry of these NCs are influenced during washing can be found by looking to the work on single-halide perovskite NCs.^28,30,31^ These include the work by Grisorio et al.,^28^ who proposed that there are interactions between polar antisolvents and the OA and OAm ligands that cause surface ligand detachment. However, detailed studies into the mixed-halide systems are needed to understand how the synergistic effects between the surface ligands and antisolvent cause the selective etching of the surface halides and how this can be mitigated. It is also critical to understand how these factors influence the optoelectronic properties of the NCs through their surface chemistry and how the purification process can be designed to maximize the PLQYs and photostability after washing.

To address these critical questions, we studied the mixed-halide system CsPbBr_*x*_I_3-*x*_, which in itself is an important composition for a variety of applications such as pure-red emitters, as well as the top cell for tandem photovoltaics with silicon.^10,32,33^ We investigated antisolvents with different polarities (relative polarity to water shown in brackets): methyl acetate (0.253), acetone (0.355), and 1-butanol (0.552), which are all commonly used for perovskite NC purification. Our studies indicate that the degree of PL blueshift and, therefore, the change in surface I-to-Br ratio is correlated to the relative polarity of the antisolvents. We integrate detailed proton nuclear magnetic resonance (^1^H-NMR), transmission electron microscopy (TEM), X-ray diffraction (XRD) and X-ray photoemission spectroscopy (XPS) analysis to understand the surface chemistry of the system. We identified that the increasing blueshift in bandgap with the polarity of the antisolvent is due to the more polar antisolvents triggering a reaction between the OA and OAm ligands, causing the removal of surface iodides. We show that these effects can be suppressed by reducing the polarity of the antisolvent. The detailed understanding of the NC purification mechanism put forward in this work could facilitate the fabrication of more stable and high-performance NCs for applications like LEDs and photovoltaics.

## Experimental Section

### Materials and Methods

#### Materials

Cs_2_CO_3_ (cesium carbonate, 99%), PbBr_2_ (lead(II) bromide, 98%), PbI_2_ (lead(II) iodide, 99%), 1-octadecene (technical grade, 90%), oleic acid (technical grade, 90%), oleylamine (technical grade, 70%), hexane (HPLC, grade ≥ 97.0%, GC), 1-butanol (HPLC, grade ≥ 97%), acetone (HPLC, ≥97%), and methyl acetate (HPLC, ≥97%). All chemicals were purchased from Sigma-Aldrich and used as received.

#### Synthesis of the CsPbBr_3_ Perovskite NCs

In a typical synthesis, 10 mL of 1-octadecene, 0.5 mL of oleic acid and 0.5 mL of oleylamine were added to Cs_2_CO_3_ (0.1 mmol) and PbBr_2_ (0.3 mmol) precursor powders. Then, tip-sonication (SONOPULS HD 3100, BANDELIN) was applied to the mixture solution at a power of 30 W for 10 min. The as-prepared NC dispersions were purified without adding any antisolvents by centrifugation at 9000 rpm for 10 min to remove unreacted precursors before redispersing the NC precipitates in 5 mL of hexane under mild sonication. The obtained NC dispersions were further centrifuged at 2000 rpm to remove large NCs. Thus, the obtained CsPbBr_3_ NCs solution is ready to undergo an anion-exchange process to prepare the CsPbBr_*x*_I_3-*x*_ NCs.

#### Synthesis of the CsPb(Br,I)_3_ Perovskite NCs

First, lead iodide precursor solution was prepared by dissolving 0.2 mmol of PbI_2_ powder in a mixture of 10 mL of toluene, 0.2 mL of oleylamine, and 0.2 mL of oleic acid at 100 °C under continuous stirring until the precursor dissolved. Then, the appropriate amount of PbI_2_-ligand solution was added to the parent CsPbBr_3_ NC colloidal solution to initiate halide ion exchange. The mixed halide ratio between Br and I is aimed to be 1:9 after the reaction. The reaction mixtures were stirred at 800 rpm for 30–40 min at 40 °C. The washing process was performed with the antisolvents of butanol, acetone, or methyl acetate. One milliliter of antisolvent was added to 1 mL of the as-prepared solution and purified by centrifugation at 8000 rpm for 10 min. The supernatant was immediately discarded, and the precipitated NCs were redispersed in 1 mL of hexane for characterization.

### Characterization

#### Ultraviolet–Visible Absorption Spectra

Ultraviolet–visible absorption (UV–Vis) spectra were recorded on a Shimadzu UV–VIS–NIR Spectrophotometer UV-3600Plus. The absorption spectra were measured on NC thin films, which were prepared by spin-coating the NC solution on precleaned glass substrates at 1500 rpm for 30 s in air. All glass substrates are cleaned with acetone and isopropanol separately in an ultrasonic bath for 15 min.

#### Steady-State Photoluminescence Spectra

Steady-state photoluminescence spectra (PL) were recorded using a Horiba Fluorolog system with an integrating sphere and a monochromatic Xe lamp as the excitation source.

#### X-ray Diffraction and Photoluminescence Quantum Yield

The crystalline structure of the perovskite films was investigated with an X-ray diffractometer (Bruker D8 Advance Powder X-ray Diffractometer) equipped with a Cu K_α_ X-ray tube using glass/perovskite as samples measured with an air-free sample holder. The PLQY of the perovskite film was measured using a commercial setup from Ocean Optics with excitation from a 405 nm wavelength continuous wave (CW) diode laser.

#### Grazing-Incidence Wide-Angle X-ray Scattering

The 2D GIWAXS data was taken at German synchrotron DESY in Hamburg, Petra III, at the P03 MiNaXS beamline.^34^ A Lambda 9M detector from X-spectrum was used with a wavelength of 1.05 Å. Processing of the 2D data was done using the software INSIGHT.^35^ This includes transformation to q-space, flatfield correction, masking, intensity corrections for detector absorption, solid angle, photon polarization, and path attenuation and obtaining pseudo-XRD cuts.

#### Fourier Transform Infrared Spectroscopy

Fourier Transform Infrared Spectroscopy (FTIR) was measured using transmission IR spectroscopy (Shimadzu IRTracer 100). The samples were prepared by spin-coating the perovskite NCs solutions washed by different antisolvents with similar concentrations (15 mg/mL) at 1500 rpm for 30 s on CaF_2_ substrates (Knight Optical) and referenced to the transmission of IR in air. The spectrum was acquired in air with Happ–Genzel apodization, a resolution of 4 cm^–1^, and 15 scans.

#### Time-Resolved Photoluminescence Spectra

Time-resolved photoluminescence spectra were obtained by exciting the perovskite films with a Pico Quant LDH407 laser diode at 407 nm with a repetition rate of 2.5 MHz at different fluences. The emission signal was selected with a monochromator to obtain the desired emission wavelength and detected by a Hamamastu R3809U-50 photomultiplier detector. Color filters (long-pass filter) were utilized to remove the scattered photons from the excitation laser.

#### Transmission Electron Microscopy and Energy-Dispersive X-ray Spectrometry

Transmission electron microscopy (TEM) and energy dispersive X-ray spectrometry (EDX) measurements were taken in STEM mode (200 kV acceleration voltage) using a FIB-TEM Helios Nanolab 450S in SAE Technologies Development (Dongguan) Co., Ltd., Guangdong Province, China. The TEM samples were prepared inside a glovebox by dropping 5 μL of NC solution with a concentration of 10 mg mL^–1^ onto a copper TEM grid and dried inside the glovebox for 20 min. A GATAN 648 vacuum transfer holder was used to eliminate the exposure to the ambient atmosphere to avoid degrading the NCs between the glovebox and TEM vacuum chamber.

#### X-ray Photoemission Spectroscopy

XPS data was acquired using a Kratos Axis SUPRA using monochromated Al K_α_ (1486.69 eV) X-rays at 12 mA emission and 15 kV HT (180 W), with an analysis area of 700 μm × 300 μm. The instrument was calibrated to the gold metal Au 4f core level (83.95 eV) and dispersion-adjusted to give a binding energy (BE) of 932.6 eV for the Cu 2p_3/2_ line of metallic copper. Ag 3d_5/2_ line FWHM at 10 eV pass energy was 0.544 eV. Source resolution for monochromatic Al K_α_ X-rays is ∼0.3 eV. The instrumental resolution was determined to be 0.29 eV at 10 eV pass energy using the Fermi edge of the valence band for metallic silver. The resolution with the charge compensation system was <1.33 eV FWHM on PTFE. High-resolution spectra were obtained using a pass energy of 20 eV, step size of 0.1 eV, and sweep time of 60 s, resulting in a line width of 0.696 eV for Au 4f_7/2_. Survey spectra were obtained using a pass energy of 160 eV. Charge neutralization was achieved using an electron flood gun with filament current of 0.38 A, charge balance of 2 V, and a filament bias of 4.2 V. Successful neutralization was adjudged by analyzing the C 1s region wherein a sharp peak with no lower BE structure was obtained. The spectra were charge corrected to the main line of the carbon 1s spectrum (adventitious carbon) set to 284.8 eV. All data was recorded at a base pressure of below 9 × 10^–9^ Torr and at room temperature (294 K). Data was analyzed using the software CasaXPS v2.3.19PR1.0.

#### Photothermal Deflection Spectroscopy

Photothermal deflection spectroscopy (PDS) is an ultrasensitive absorption measurement technique that detects heating of the sample due to the nonradiative relaxation of absorbed light and is insensitive to reflection and scattering. PDS enables the detection of absorbance signals with 5–6 orders of magnitude weaker than the band edge absorption.^36^ For the measurements, the sample (film on quartz substrate) was illuminated with a monochromatic pump beam. Light absorption leads to a thermal gradient near the sample surface via non-radiative relaxation induced heating. This results in a refractive index gradient in the area surrounding the sample surface. This refractive index gradient is further enhanced by immersing the sample in an inert liquid FC-72 Fluorinert (3M Company), which has a high refractive index change per unit change in temperature. A fixed wavelength cw laser probe beam was passed through this refractive index gradient, producing a deflection proportional to the absorbed light at that particular wavelength, which is detected by a photodiode and lock-in amplifier combination. Scanning through different wavelengths gives us the complete absorption spectra. Because this technique makes use of the non-radiative relaxation processes in the sample, it is immune to optical effects like interference and scattering.

#### Proton Nuclear Magnetic Resonance

Room temperature liquid-state ^1^H NMR (500.2 MHz) spectra were recorded on a Bruker Avance III HD 11.7 T spectrometer equipped with a BBO Smart Probe. ^1^H chemical shifts were referenced using the residual CH_3_ signal of toluene-*d*_6_ (2.51 ppm) as a secondary reference. All NMR samples were prepared by adding 5 μL of test samples (including original NC solution, different-antisolvent washed NC solution, and corresponding supernatants oleic acid, oleylamine, and different antisolvents) into 5 mL of toluene-*d*_6_.

## Results and Discussion

### Influence of Antisolvent Polarity on NC Optoelectronic Properties

**Figure 1:**
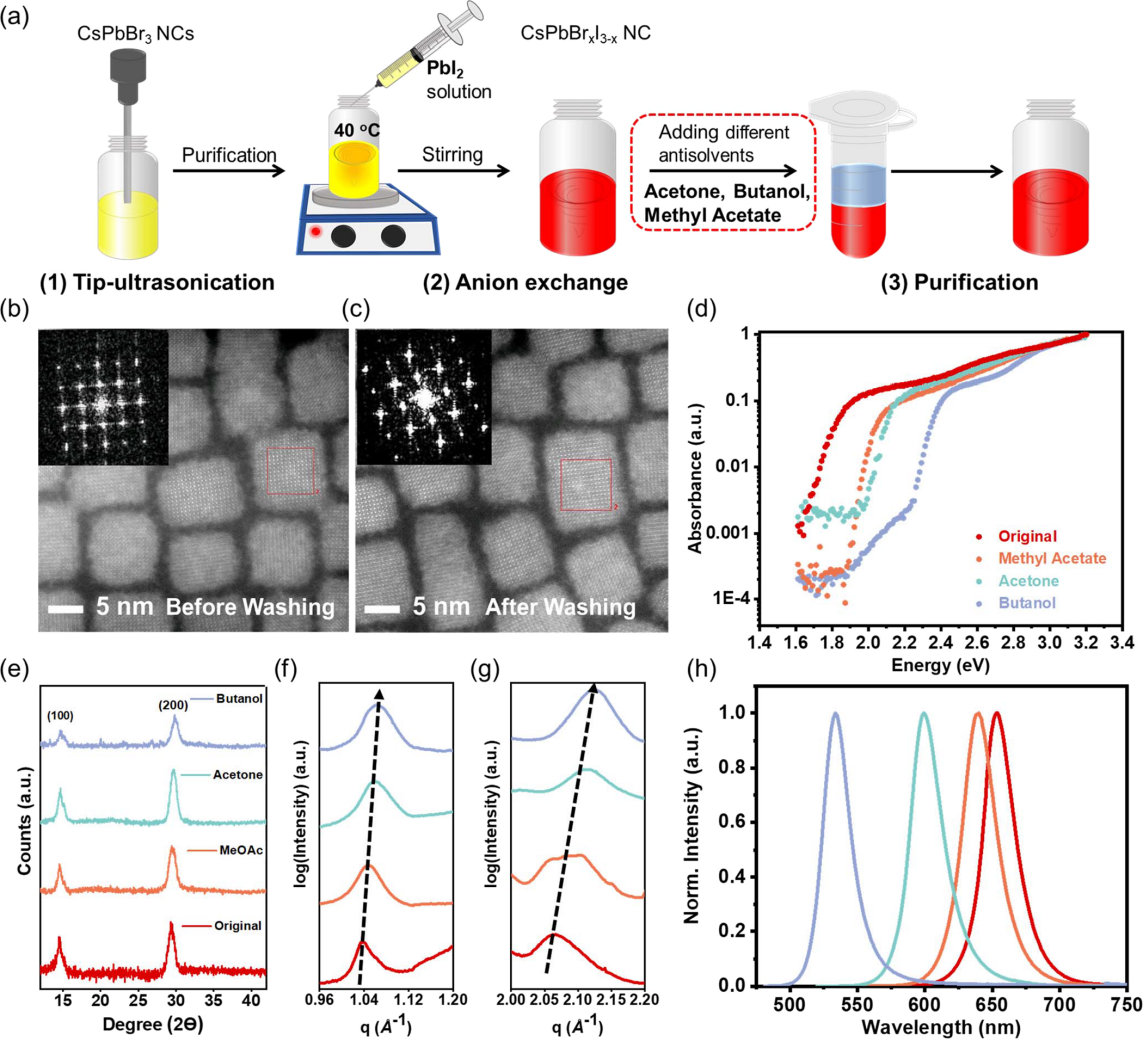
(a) Schematic illustration of the synthesis of mixed bromide-iodide inorganic perovskite nanocrystals (NCs) through halide exchange on CsPbBr_3_ NCs, which were grown by a tip-sonication approach. The anion exchange reaction replaces bromides with iodides to obtain red-emitting CsPbBr_*x*_I_3-*x*_ NCs, which are purified by adding different antisolvents, including methyl acetate, acetone, and 1-butanol. (b,c) Scanning Transmission Electron Microscopy (STEM) of the as-synthesized (left) and acetone-washed CsPbBr_*x*_I_*3-x*_ NCs (right). Inset contains the fast Fourier transform images of the NCs in the selected red region. (d) Photothermal deflection spectra of the pristine and washed NC films. (e) X-ray diffraction patterns of pristine and purified NC thin films on glass substrates. (f) GIWAXS line cut plots for (100) Bragg reflex in q space. (g) (200) Bragg reflex in q space. (h) Photoluminescence spectra of CsPbBr_*x*_I_*3-x*_ NC thin films after purification with different antisolvents: (red) no antisolvent (dissolved in hexane only), (yellow) methyl acetate (MeOAc) (1:1, v/v); (green) acetone ((1:1, v/v) ;(blue) butanol (1:1, v/v).

**Figure 2:**
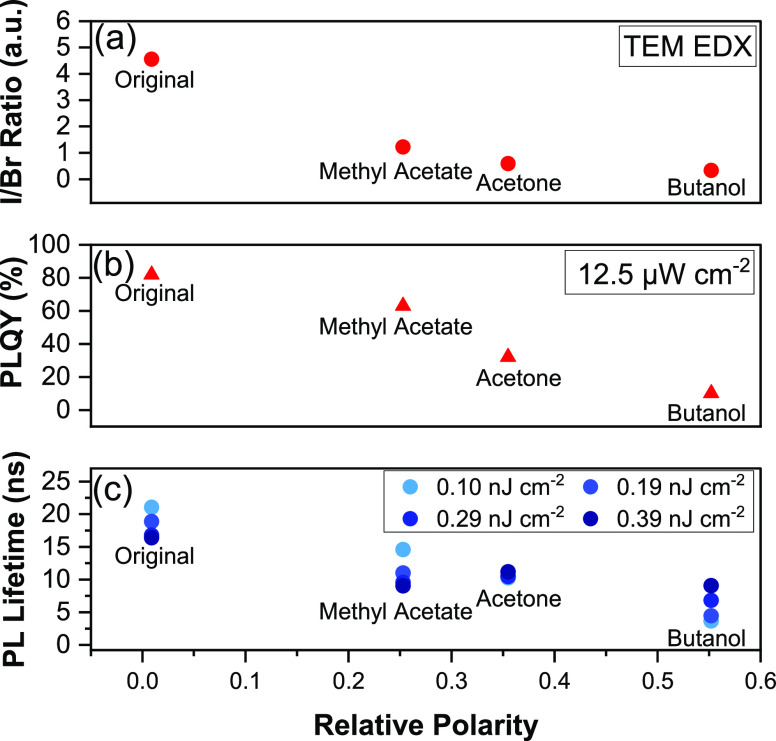
Influence of the relative polarity of the antisolvent on the (a) I/Br ratio, (b) PLQY, and (c) PL lifetime of the washed NCs as compared to the NCs precipitated without the use of any antisolvents (denoted as a relative polarity of 0).

The mixed I-Br halide perovskite nanocrystals (CsPbBr_*x*_I_3-*x*_ NCs) were synthesized by tip-ultrasonication, followed by anion exchange, as previously reported by Tong et al.^37,38^ The synthesis scheme is shown in Figure 1a, with the scanning transmission electron microscopy (STEM) image of the NCs shown in Figure 1b and Figure S1, SI. The as-prepared NCs were washed in different antisolvents to remove unreacted precursors (Figure 1a, step 3). The obtained NCs were redispersed in hexane and spin-coated onto glass substrates for characterization. Based on the photothermal deflection spectroscopy and UV–Vis absorption spectroscopy measurements (Figure 1d and Figure S2, SI), the absorption of the original NC thin films is blueshifted after washing with more polar antisolvents, from methyl acetate to acetone to butanol. The original as-prepared CsPbBr_*x*_I_3*-x*_ NCs had an absorption onset at ca. 630 nm and additional absorption peak at 560 nm (Figure S2, SI). The dual absorption peak in the crude original solution may originate from unreacted precursors. After washing with different antisolvents (acetone, 1-butanol, or methyl acetate), the absorption peak at 560 nm disappeared, likely due to the removal of these excess precursors. XRD and EDX measurements showed that the NCs remained as perovskites after purification. This is because the diffraction peaks after washing matched those of the cubic perovskite phase (Figure 1e). Also, no significant differences in the elements present and their distribution across the NCs were observed in the elemental mapping measurements, which showed that all NCs after washing still retained Cs, Pb, I, and Br (Figure S4, SI). However, by washing with different antisolvents, we observed a shift in the absorption onset in the PDS spectra (Figure 1d), along with a concomitant shift in PL peak position (Figure 1h). With no solvent used during washing (i.e., by centrifuging only), the PL peak of the resulting NCs was centered at 653 nm. With the use of methyl acetate, acetone, or butanol during purification, the PL peak blueshifted to 638, 599, and 533 nm, respectively (Figure 1h and Table 1). In addition to these changes in the bandgap, the PLQY and PL lifetime (measured from spin-coated films of NCs) also decreased after washing (Table 1 and Figure 2b,c). We observed that these decreases were larger when more polar antisolvents were used. To understand these observations, we first determined whether quantum confinement effects could have played a role (i.e., if the NC size reduced after washing). We analyzed the distribution in the sizes of the NCs measured by TEM before and after washing with different antisolvents (Figure S1, SI) and found there to be no significant changes (Figure S5, SI). An example can be found in Figure 1b,c, which compares the original NCs and NCs after washing with methyl acetate. Furthermore, the median size of the NCs in all cases was close to 8 nm (Figure S5, SI). These results therefore rule out the possibility of size-induced changes in PL emission wavelength.

**Table 1:** Properties of Antisolvents and their Influence on the Optoelectronic Properties of CsPbBr_*x*_I_3 – *x*_ NCs^a^

washing solvent	solvent polarity	dielectric constant (298 K)	solvent DN/AN	PL peak (nm)	FWHM (nm)	PLQY (125 μW cm^–2^)	I composition from TEM EDX (XPS)
hexane	0.009	1.9	0/0	653	35	81.9%	82 at %
methyl acetate	0.253	6.7	16.5/10.7	638	35	62.9%	55 at % (44 at %)
acetone	0.355	20.7	17.0/12.5	599	32	32.1%	37 at % (18 at %)
butanol	0.552	17.3	19.5/36.8	533	34	10.1%	25 at % (14 at %)

a The PLQY was measured using a 405 nm cw laser under different power densities. DN = Gutmann donor number, AN = Gutmann acceptor number. Dielectric constant is the average dielectric constant of the antisolvents. Solvent polarity is the relative polarity to water.

On the other hand, TEM-EDX and XPS results showed that the NC chemical composition in both the bulk and surface became more bromide-rich after washing (Table S1 and Figure S12, SI), especially with the use of more polar antisolvents. This relationship between the antisolvent polarity and Br/I composition and PL properties is shown in Figure 2. Figure 2a demonstrated a clear inverse relationship between the antisolvent polarity and the I/Br ratio. Washing with more polar antisolvents tends to reduce the I/Br ratio more as the surface I was removed. This is also observed in the I 3d core level XPS spectrum shown in Figure S12a, SI, in which the I 3d XPS peak intensity decreased more after washing with butanol than methyl acetate or acetone. In addition, XRD measurements showed that the (100) and (200) peaks of the NCs shifted toward higher 2θ values as the antisolvent polarity was increased (Figure 1e), showing that the lattice parameter was reduced, which correlates with the removal of the larger I^–^ anions. The calculated lattice constants are shown in Table S2, SI. The calculated lattice constant decreased from 6.11 Å (original) to 6.08 Å (methyl acetate-washed), to 6.03 Å (acetone-washed) and 6.02 Å (butanol-washed). The calculated value is in between 6.02 Å (cubic CsPbBr_3_) and 6.41 Å (cubic CsPbI_3_).^39^ The change in d-spacing is also observed in our GIWAXS line cut plot. (Figure 1f,g, and Figures S6 and S7, SI). Figure 1f,g shows that the *q*-value increased for the (100) and (200) planes as the NCs were washed with more polar solvents, which is consistent with the 1D XRD θ–2θ line scan results shown in Figure 1e. Overall, these observations suggest that increasing the polarity of the antisolvent led to the preferential removal of I, which in turn leads to an increase in the density of non-radiative recombination centers that reduce the PLQY. This is also confirmed by PDS measurements (Figure 1d), which show an increase in the sub-bandgap absorption of the colloidal NC solution after washing with the most polar antisolvents (acetone or butanol). In addition, the absorption onset became less steep after washing with more polar antisolvents, indicating an increase in disorder (see Figure S3 and Table S3, SI). The methyl acetate-washed NC has the steepest slope, which indicates that it has less disorder as the methyl acetate-washed NCs have the unreacted ligands and precursors removed without creating too many surface defects. The increased disorder (acetone and butanol washed NCs) may arise as a consequence of the selective removal of iodide species from different parts of the NCs during purification.

To understand the influence of these defects introduced after washing on the PL lifetime, TCSPC was measured on the spin-coated NC films. The data is fitted with a numerical three-component exponential decay function shown in Table S4, SI. We note that while this model is not necessarily physically relevant, it provides a numerical description of the PL decay curves that is not reliant on knowing the recombination mechanism a priori or fitting a large number of variables to each PL decay curve, which may lead to inaccurate local minima fittings. Figure 2c and Table S4, SI, show the fitted average time constants from our model at different fluences from 0.009 nJ cm^–2^ pulse^–1^ to 0.39 nJ cm ^–2^ pulse^–1^. At low fluence (0.009 nJ cm^–2^ pulse^–1^), the PL lifetime decreased from 21.05 ns (original) to 14.58 ns (methyl acetate), 10.25 ns (acetone) and 3.67 ns (butanol), as shown in Figure S8a, SI. The decrease in PL lifetime after washing with more polar solvents (Figure 2c) is consistent with the presence of a high density of recombination centers. This trend of decreasing in PL lifetime shown in Figure S8a also matches the decreasing trend of PLQYs as shown in Figure 2b. However, as we increased the fluence, the PL lifetime decreased for the original NCs, as well as the NCs washed with methyl acetate (Figure S8e,f, SI). Since the measurement fluence is in the nJ cm^–2^ pulse^–1^ range, Auger recombination is unlikely to have occurred. In low defect-density systems, (i.e., the original NCs and methyl acetate-washed NCs), the decrease in PL lifetime when increasing the measurement fluence could be explained by an increasing proportion of radiative recombination (bimolecular) as opposed to Shockley–Read–Hall recombination. In contrast, the PL lifetime increased with increasing excitation fluence for the acetone- and butanol-washed NCs (Figure S8g,h, SI). The increase in PL lifetime is likely due to the trap-filling effect as the fluence increased.^40^ These washing-induced defects can be atomically resolved with HAADF-STEM images and are visualized in Figure S9, SI. The HR-TEM images of the NCs (Figure S9, SI) provides several pieces of evidence for structural defects in the purified NCs. Figure S9a,b shows a single acetone-washed NC, in which the bottom half of the NC is maintained as a cubic-phase perovskite, but the top half has been changed to the Ruddlesden–Popper (RP) phase. In the RP phases, all the [001]-projected cation atom columns are composed of Cs–Pb–Br columns (Figure S9a,b).^41^ This matches with the previous study by Yu et al., in which they also observed the coexistence of cubic- and RP-phase perovskites in CsPbBr_3_ nanosheets.^41^ The boundary between the two phases may be a site for non-radiative recombination to quench the PL. Another form of defects observed in the washed NCs are line defects, as shown in Figure S9c–e, SI. There are clear line cuts on the same NCs, which separate the perovskite into two regions with about 1/2 lattice offset (shown by the region surrounded by the red dotted lines in Figure S9d,e). We believe that this is a typical antiphase boundary, and it is regarded as a translation boundary defect in the perovskite phase.^41,42^ Last, Figure S9f shows a change in brightness on the same NCs after washing, indicating that there is either a change in the thickness or a change in the surface termination in these dark and bright regions.^41^ All of these atomic-level structural defects, along with point defects, could act as non-radiative recombination centers that influence charge-carrier dynamics. As these traps occurring at these structural defects were increasingly filled at higher fluences, and fewer were available for trap-mediated recombination, leading to an increase in the PL lifetime as shown in Figure S8g,h.

### Mechanistic Study into Antisolvent Polarity-Induced Changes in the Surface Chemistry of LHP NCs

**Figure 3:**
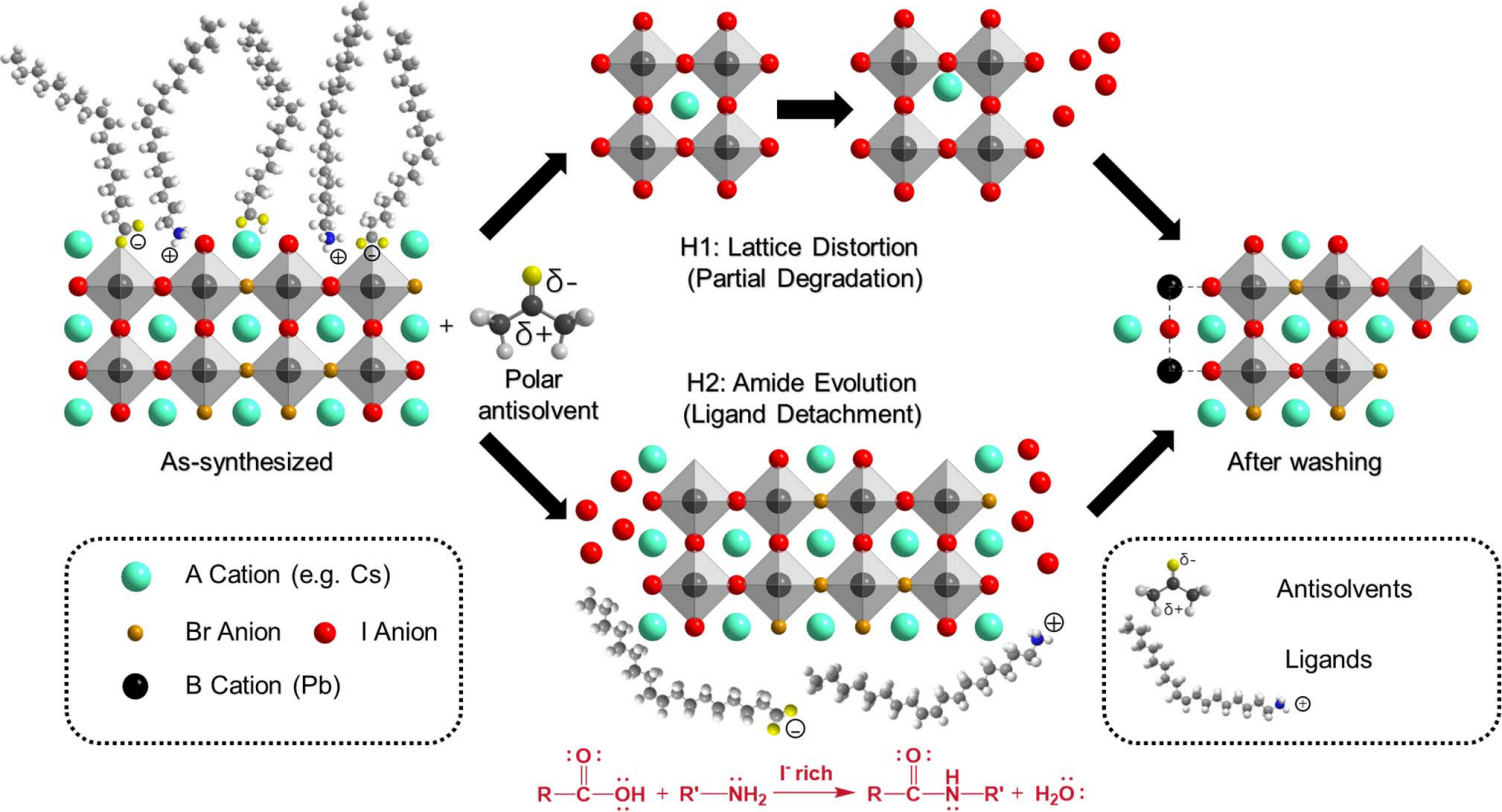
Proposed mechanisms for the antisolvent-dependent selective etching of the mixed I-Br perovskite NCs. Hypothesis one (H1): Solvent-induced lattice distortion, which leads to nanocrystal degradation and the formation of PbI_2_. Hypothesis two (H2): Polar solvent-induced amide evolution, which leads to surface ligand and iodide detachment.

To explain the cause of the dependence of the composition and optoelectronic properties of CsPbBr_*x*_I_3-*x*_ perovskite NCs on the polarity of the antisolvent during purification, we propose two potential mechanisms as illustrated in Figure 3. Here, we will first detail these two mechanisms before providing experimental evidence for or against them.

Our first hypothesis is antisolvent-induced lattice distortion. Antisolvents with different polarities could impose different lattice distortions on the NCs during washing. When more polar antisolvent molecules (e.g., acetone) interact with the iodine-rich NCs (original NCs have a Br/I ratio of approximately 18%:82%), the strong dipole present in the antisolvent molcule causes a distortion in the [PbI_6_]^4–^ octahedra on the surface. Sun et al. previously found from density function theory (DFT) calculations that when polar ethanol molecules are chemisorbed onto the surface of CsPbI_3_ NCs, the polar molecule can induce a shift in the electron cloud of the Cs^+^ cations, thus breaking the symmetry of the surrounding [PbI_6_]^4–^ octahedra and inducing polarization in CsPbI_3_.^43^ The distortion could lead to perovskite NCs degrading into PbI_2_ during centrifugation, which could then cause an overall loss in iodide content and defect formation at the surface of the NCs. However, the proposed mechanism requires the antisolvent to be in direct contact with the NC surface. By contrast, there is strong evidence that the NC surface is capped by organic ligands. XPS measurements (Figure S12a, SI) support this because the intensity of the I 3d core level of the original NCs are very low compared to the purified NCs despite the original NCs having higher I content as found from TEM EDX. By having a high ligand density, the I signal in XPS is obscured by the long carbon-based chains. Additionally, our DFT calculations show that the oleylammonium ions bind with surface halides through hydrogen bonding. Meanwhile, oleate ions balance the NC surface charge without forming bonds (Figure S10, SI). This result is consistent with previous solid-state NMR analysis by Chen et al.^31^ Since the NCs are capped with the ligands, they will protect the NCs from substantial contact with the antisolvents. The antisolvent molecules are likely to be sterically hindered from interacting with the NC surface due to the presence of ligands. Furthermore, we found that adding excess PbI_2_ to all colloidal solutions of purified NCs resulted in the PL peak redshifting and stabilizing at a wavelength of 680 nm (Figure S11, SI). One possible explanation would be that the excess PbI_2_ can reconstruct the damaged lattice and restore it to its state before purification (i.e., the I ratio increased and PL peak redshifted). However, we cannot rule out other possibilities such as the added PbI_2_ triggering further halide exchange between the exposed surface Br after purification with the added iodides. To further test the validity of hypothesis 1 (H1, lattice distortion), we investigated the surface composition of the washed NCs with XPS (Figure S12, SI). We found that the intensity of the Pb 4f core level spectrum remained almost the same for NCs washed by different antisolvents, whereas there was an obvious reduction in the I 3d core peak intensity after washing with more polar antisolvents, indicating that perovskite degradation to PbI_2_ is unlikely as only the I content changed while the Pb content remained almost the same. We also examined the elemental composition of the supernatants following washing using SEM–EDX (Figure S17b–d, SI). The ratio of I/Br in the supernatants showed the opposite trend to that found in NCs (measured by TEM-EDX; Figure 2a). In the supernatants, the I ratio increased with antisolvent polarity, indicating that more I was removed from the NCs (Figure S17a, SI). Similarly, the Pb ratio in the supernatants remained approximately constant (∼29%; Figure S17a, SI), which was consistent with the XPS results (Figure S12, SI). Therefore, we believe that perovskite degradation is unlikely to be the primary reason for iodide removal when washing with polar antisolvents, since only the I content changed significantly. The addition of excess PbI_2_ would repair the halide vacancies induced during the washing process or undergo halide exchange to reintroduce extra I to the surface of the NCs.

**Figure 4:**
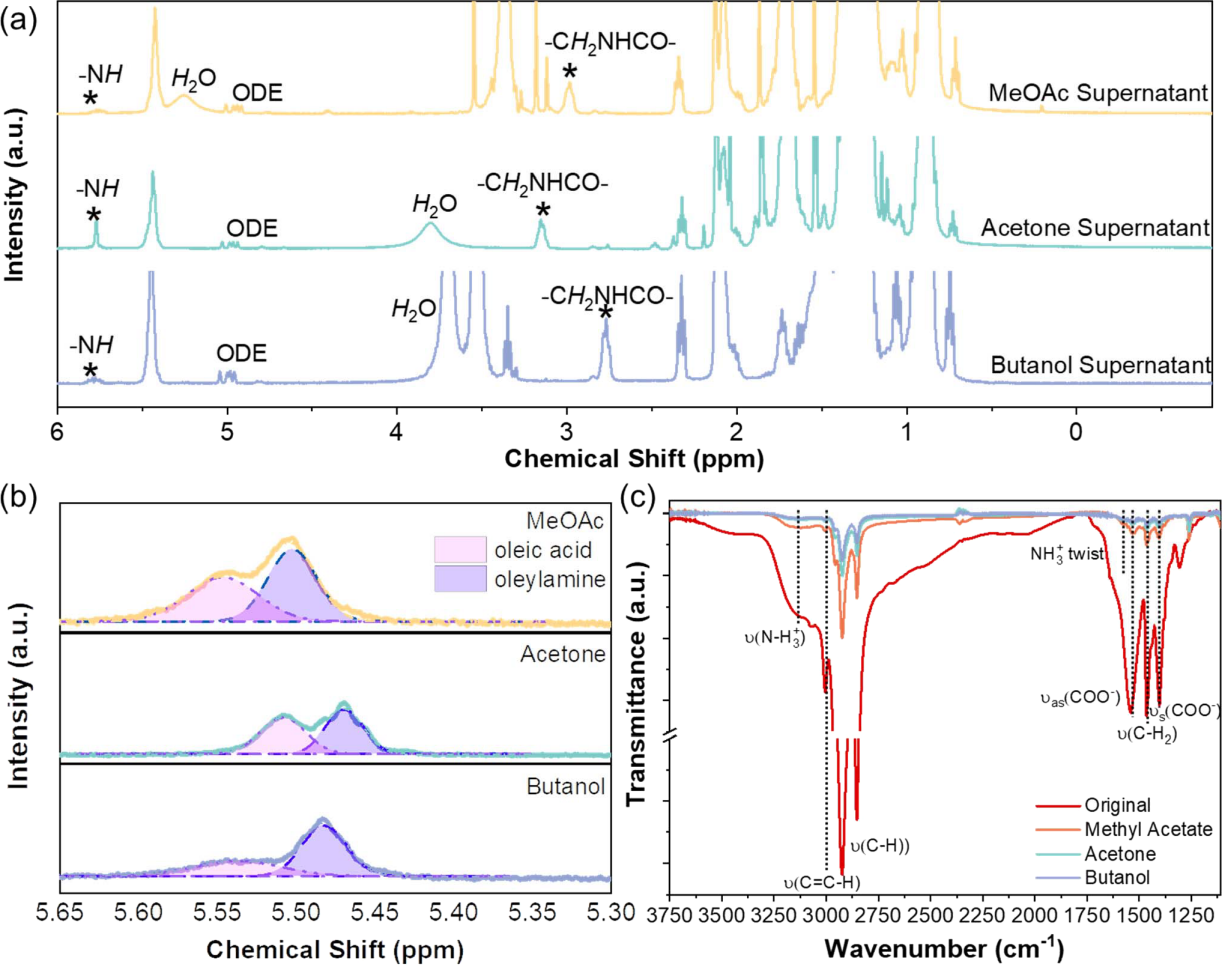
(a) ^1^H NMR spectra of the supernatant solutions after NC purification. (b) Close up of the C=C double-bond section of the ^1^H NMR spectra for oleate and oleylammonium ligands on washed NCs. (c) FTIR transmittance spectra of spin-coated NC films on CaF_2_ substrates.

Our second hypothesis is antisolvent-induced amide evolution that leads to the detachment of surface ligands and iodides. Initially, NCs are stabilized in their original colloidal solution with unreacted precursors, including PbI_2_-ligand, Cs- or Pb-oleate, ammonium-halide (R-NH_3_^+^-Br^–^/I^–^), and unbound ligands (R-COOH and R-NH_2_). The initial NC surface is iodine-rich and capped by oleic acid (R-COO^−^) and oleylammonium (R-NH_3_^+^) ligands. During the washing process, the addition of the polar solvents destroys the original charge balance of ligands bound to the surface of the NCs, triggering condensation reactions between the amine groups from the oleylammonium ligands and carboxylic acid groups from oleate ligands to form amide groups on an I-rich NC surface. The formation of amides will likely lead to a passivation failure where the amine and carboxylic acid groups can no longer bind with the NC surface to protect the NCs from the surroundings. This is consistent with the decrease in FTIR transmittance (at 3300–2500 cm^–1^) of the signature peak for oleic acid ligands (Figure 4c). As the ligands are removed, the NCs are exposed to the surrounding polar molecules. The polar solvents may easily attach to the surface of the NCs and strip away the surface iodine through this amide evolution reaction. As a result, the Br/I ratio will be changed until a new chemical equilibrium is re-established when the Cs surface or uncoordinated Pb atoms are repassivated by the ligands in solution. XPS measurements (Figure S12b,c, SI) show that the Br 3d and Pb 4f core level peaks are not significantly influenced by washing with different antisolvents. It is only the I 3d core peaks that decrease in intensity after washing. These results suggest that it is primarily the I species that are influenced by the polar solvents, which is in good agreement with the TEM-EDX (Table S1, SI) measurements of the bulk composition of the NCs. We believe that the reason for the Br species to not be as strongly influenced by the polar solvents is that the mixed-halide NCs we studied in this work were prepared through halide exchange from CsPbBr_3_ as the starting material. The diffusion-driven halide exchange process will exchange the outer Br with I first.^44^ As a result, the outside of the NCs during washing would be iodine-rich, and as I is a larger atom than Br, it is unlikely for I to diffuse to the inner core of the NCs.^28^ Therefore, the Br, present in high concentrations in the inner part of the NCs, are not as significantly affected as the I during the purification process, which possibly explains the unchanged Br 3d core-level spectra in Figure S12b, SI.

To further understand the effect of antisolvents on the selective etching of surface halides relevant to the initial surface halide composition, we examined the PL of two new systems in contrast to the results described above on CsPbI_3-*x*_Br_*x*_ NCs prepared by diffusing I into CsPbBr_3_. The first system is mixed-halide NCs prepared by diffusing Br into CsPbI_3_ NCs (i.e., PbBr_2_ was added to CsPbI_3_ NCs solutions to trigger the Br halide exchange as shown in Figure S16a,b, SI. Interestingly, the PL peak was still blueshifted after purifying these CsPbBr_*x*_I_3-*x*_ NCs. Figure S16b shows that with different amounts of PbBr_2_ added to CsPbI_3_ NCs, the PL peak positions of the unwashed NCs can be tuned to represent different initial Br/I compositions (e.g. 605 nm (high I content), 575 nm (moderate I content), and 537 nm (low I content)), we found that the amount of blueshift in PL after washing is related to the initial Br/I ratio (larger blueshifts were observed if the initial I content was higher; Figure S16b, SI). Similarly, the extent of blueshift was smaller than found from mixed-halide NCs obtained by diffusing I into CsPbBr_3_ (Figure 1h, Table 1, and Table S5), which is likely due to the less effective removal of I ions due to the Br-rich surface. The results suggest that the antisolvents etch the I ions more effectively compared to Br ions, even though we have halide exchange from the I-rich surface to the Br-rich surface. The second system examined is CsPbBr_3_ NCs, i.e., where the surface halide is purely Br. A similar phenomenon was observed by washing the CsPbBr_3_ NCs with antisolvents of different polarity. The PLQY decreased from 55.0% for the original CsPbBr_3_ NCs to 32.2% for NCs washed with methyl acetate, 28.0% for NCs washed with acetone, and 19.1% for NCs washed with butanol, as shown in Figure S16c, SI. This time, there was no change in PL peak position since there was only one halide species (Figure S16c, SI, inset). However, XPS measurements of the CsPbBr_3_ NCs showed a reduction in the ratio of Br:Pb after washing with methyl acetate (Figure S16d, SI). The relative ratio of surface Br decreased from 42 to 39%, while the ratio of surface Pb increased from 58 to 61%. This is consistent with a removal of surface Br, which correlates with the decrease in PLQY found. Therefore, these results show that it is the surface halides that are primarily affected by the removal of the ligands and that the selective etching of I occurs more easily than Br removal.

**Figure 5:**
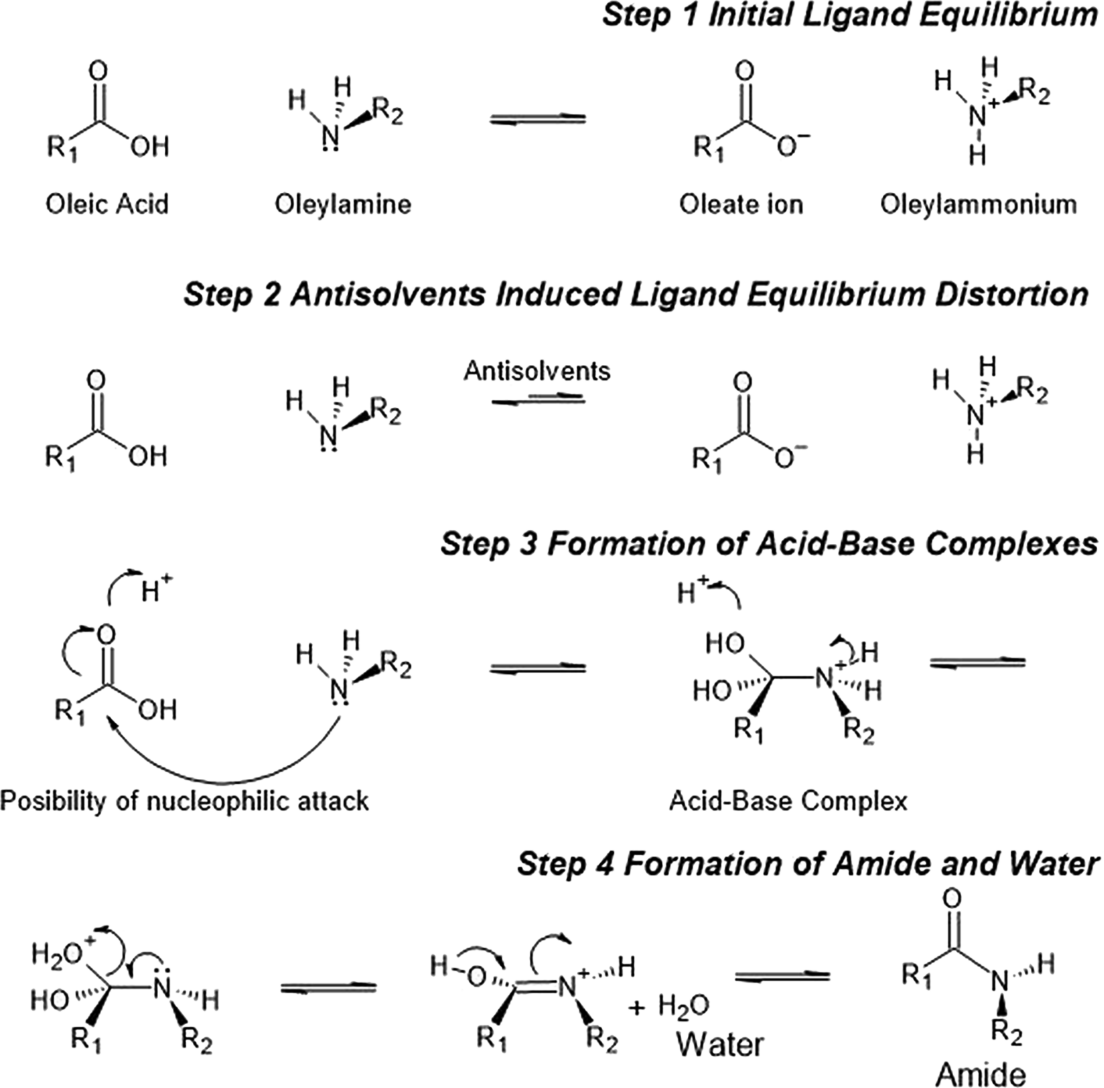
Mechanism of amide formation for Hypothesis 2 from the reactions between oleylammonium and oleate ligands on the surface of the NCs.

The proposed mechanism of amide formation is summarized in Figure 5. In the original NC solution, the oleic acid and oleylamine ligands achieve a dynamic equilibrium with their ionic forms: oleate and oleylammonium ions (Figure 5, step 1). When antisolvents are introduced into the solution, the polar antisolvent molecules would distort the original equilibrium (Figure 5, step 2). The presence of polar antisolvent molecules triggers the amine–carboxylic acid condensation reaction at the surface of the NCs.^28,45^ The interactions between the acidic ions and polar antisolvents allow the attack of the lone pair electrons and transfer from the nitrogen nucleophile in the amine group to the carbon in the carboxylic acid group (Figure 5, step 3). Intermediates are then formed through the deprotonation and protonation of the carbonyl group to form an acid–base complex (Figure 5, step 3). The direct proton transfer to the hydroxyl group in the intermediates allows the formation of charged -OH^2+^ groups, which are subsequently removed from the complexes as a neutral water molecule by-product (Figure 5, step 4). Last, as the water molecule is removed, amides are formed as the final products (Figure 5, step 4). Once the amide group is generated, the ligands lose the ability to bind to the surface as both the ammonium and carboxylic acid groups are consumed to form amide groups. As the ligands are detached, the iodides that were previously bound to the ammonium groups are also removed. Typically, amide formation requires heating at above 100 °C.^46^ However, here, we believe that the condensation reaction can occur at room temperature in the presence of polar antisolvents because the perovskite NCs could act as catalysts. Based on the available evidence in the literature,^28,47^ the catalytic effect of the perovskite NCs is likely achieved by (1) the close proximity of the amine and acid groups from ligands bound to the surface of the NCs and (2) the release of oleylammonium ligands resulting in excess protons being introduced to the NC surface, which can interact with the carboxylic acid groups from the oleic acid ligands, rendering them more amenable to attacking the amine groups (see Figure 5, especially steps 2 and 3).^47^ Similarly, it has previously been shown that amides can form at room temperature when appropriate catalysts are added, such as boronic acids or SO_2_F_2_.^48,49^ Amine–acid ligand reactions have also been observed in NaYF_4_ nanoparticles with oleic acid and octadecylamine ligands at room temperature.^45^

To determine the validity of this second hypothesis, ^1^H NMR measurements were performed on both the colloidal solutions of the washed NCs, as well as the supernatant solutions (Figure S13, SI). Here, the NMR samples were prepared by adding 5 μL of pure oleic acid, oleylamine, antisolvents, the as-synthesized and purified NCs and their corresponding supernatants solutions individually into 5 mL of deuterated toluene (C_6_D_5_CD_3_). The spectra can be found in Figure S13, SI. By comparing the known ^1^H-NMR peaks from the pure oleic acid and oleylamine with those found from the supernatant solution, we identified some new species, including peaks at δ = 2.98 ppm and δ = 5.25 ppm for methyl acetate washed NCs, δ =3.15 ppm; δ = 3.80 ppm for acetone washed NCs, and δ = 2.76 ppm and a significant increase for the peak at δ = 3.72 ppm for butanol washed NCs (shown in Figure 4a). The peaks at δ = 2.98, 3.15, and 2.76 ppm can be assigned to the hydrogen atoms (italic) in the −C*H*_2_NHCO– group.^28^ The peaks at δ = 5.9 ppm are likely to be the signal from −N*H* protons in the amide. The broad peaks at δ = 5.25, 3.80, and 3.72 ppm are attributed to protons in the OH group, which can be due to water molecules (Figure 4a). The NMR spectra are normalized to the aromatic signals of the residual protonated toluene (Figure S18, SI). The toluene here is referred to as residual because the solvent is nearly fully deuterated. Since each sample examined by NMR comprised the same amounts of solvent and test sample (i.e., 5 mL of *d*_6_-toluene + 5 μL of supernatant) and the way of obtaining the NCs and their supernatants were also identical, it is reasonable to compare the relative peak intensities by normalizing the signals to the reference aromatic signals at 7.11–7.09 ppm (Figure S18, SI). The results show that there are indeed more amides being produced if we washed with higher-polarity antisolvents as the peak intensity for the −C*H*_2_NHCO– group is the highest in the supernatants obtained after butanol washing and lowest in the supernatants obtained after methyl acetate washing. We note that chemical shifts can be strongly affected by the solvent. We therefore attribute the differences in chemical shifts of these peaks to differences in polarity of the solvents. Overall, the peak positions show good agreement with the results from Grisorio’s work.^28^

Apart from the formation of amide and water, another observation that supports the second hypothesis is that the change in the ratio of the surface ligands (oleate and oleylammonium) after washing is antisolvent-dependent, as shown in the ^1^H NMR (Figure 4b) and FTIR spectra (Figure 4c). From the NMR spectra, the peaks at δ = 5.55, 5.51, and 5.54 ppm are associated with vinyl groups (C=C) in oleate ions, while those at δ = 5.51, 5.47, and 5.48 ppm are associated with vinyl groups in oleylammonium ions for NCs washed with methyl acetate, acetone, and butanol respectively.^28^ These are ligands remaining on the surface of NCs after purification (Figure 4b), and we found that the ratio of OA to OAm surface ligands was highest when using the low-polarity methyl acetate, and lowest ratios were found when using the most polar butanol. When using acetone, this ratio remained fairly similar to the NCs washed with methyl acetate. From the amide evolution hypothesis, OA and OAm should be lost in equal amounts. However, we can see that there were slightly more OAm than OA. It is therefore possible that with butanol washing, a sufficiently large quantity of OA and OAm was removed that the NMR trace was dominated by the residual excess OAm. Indeed, from FTIR (Figure 4c), the signature peak (O–H stretch) for the carboxylic acid group in the oleic acid ligands (3300–2500 cm^–1^) reduced more as the antisolvent polarity increased. Similarly, the stretching mode of N-H_3_^+^ from 3250–3100 cm^–1^ and the twisting mode of N-H_3_^+^ at 1580 cm^–1^ in oleylammonium ions in Figure 4c demonstrated the same trend, indicating that more oleylammonium ligands are removed as antisolvent polarity is increased. These results support the second hypothesis by showing that more surface ligands take part in the amide formation reaction when using more polar antisolvents and fewer ligands are maintained on the surface of the NCs after washing. As a result, more surface iodide anions bound to the ligands are removed when using acetone and butanol.

We note here that while the data more strongly supports the second hypothesis, we cannot rule out the possibility that the first proposed mechanism occurs after the ligands are removed during the condensation reaction. Therefore, the loss of iodides may be due to both amide evolution and polar solvent-induced NC degradation after some of the surface ligands are detached.

### Impact of Antisolvents on the Stability in Emission Wavelength from NCs under the Application of an Electric Field

**Figure 6:**
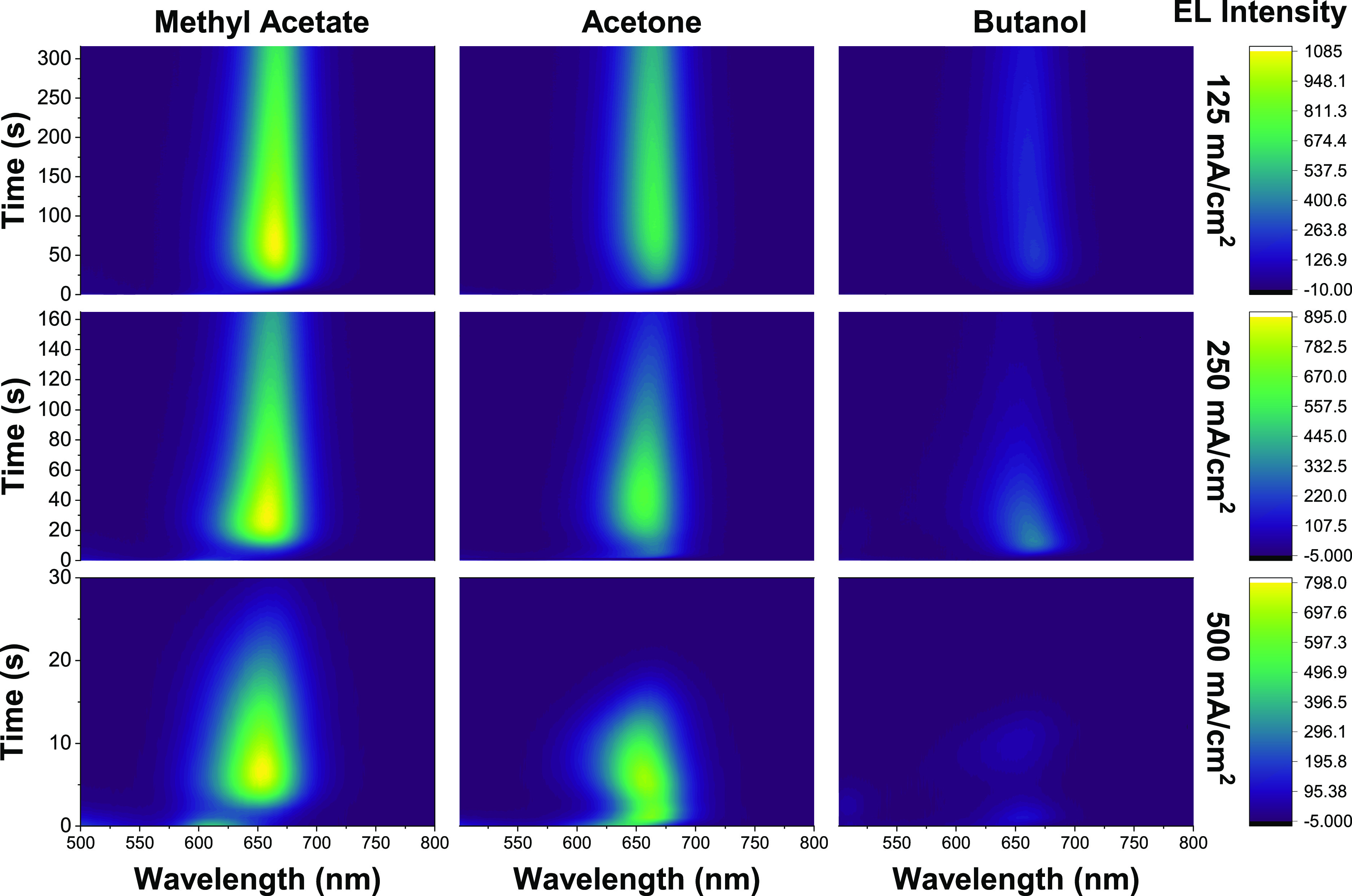
Electroluminescence stability of the purified NC-based LEDs, in which the CsPbBr_*x*_I_3-*x*_ NCs were purified using methyl acetate, acetone, or butanol antisolvents.

So far, we have discussed the influence of the antisolvents used during purification on the NC surface chemistry and optical properties. Having shown the impact of the antisolvent polarity on the density of iodide vacancies in the NCs, we investigate the implications on the stability of the emission wavelength. It is well known that halide vacancies in perovskites act as enablers for ion migration, and this can accentuate the phase segregation of mixed-halide perovskites. For LEDs, phase segregation can occur when an applied bias is used, which can cause the electroluminescence (EL) peak to redshift to the lowest-bandgap species formed. Having understood how to control the iodide vacancy concentration after purification, we elucidated the impact on EL stability. Our LED test structures had the following architecture: glass/ITO/PEDOT:PSS/Poly-TPD/perovskite NC/TPBi/LiF/Al. The device fabrication procedure can be found in the Supporting Information. Figure 6 and Figure S14, SI, demonstrate the change in EL spectra over time for devices made by NCs washed with different antisolvents and current injection levels.

**Figure 7:**
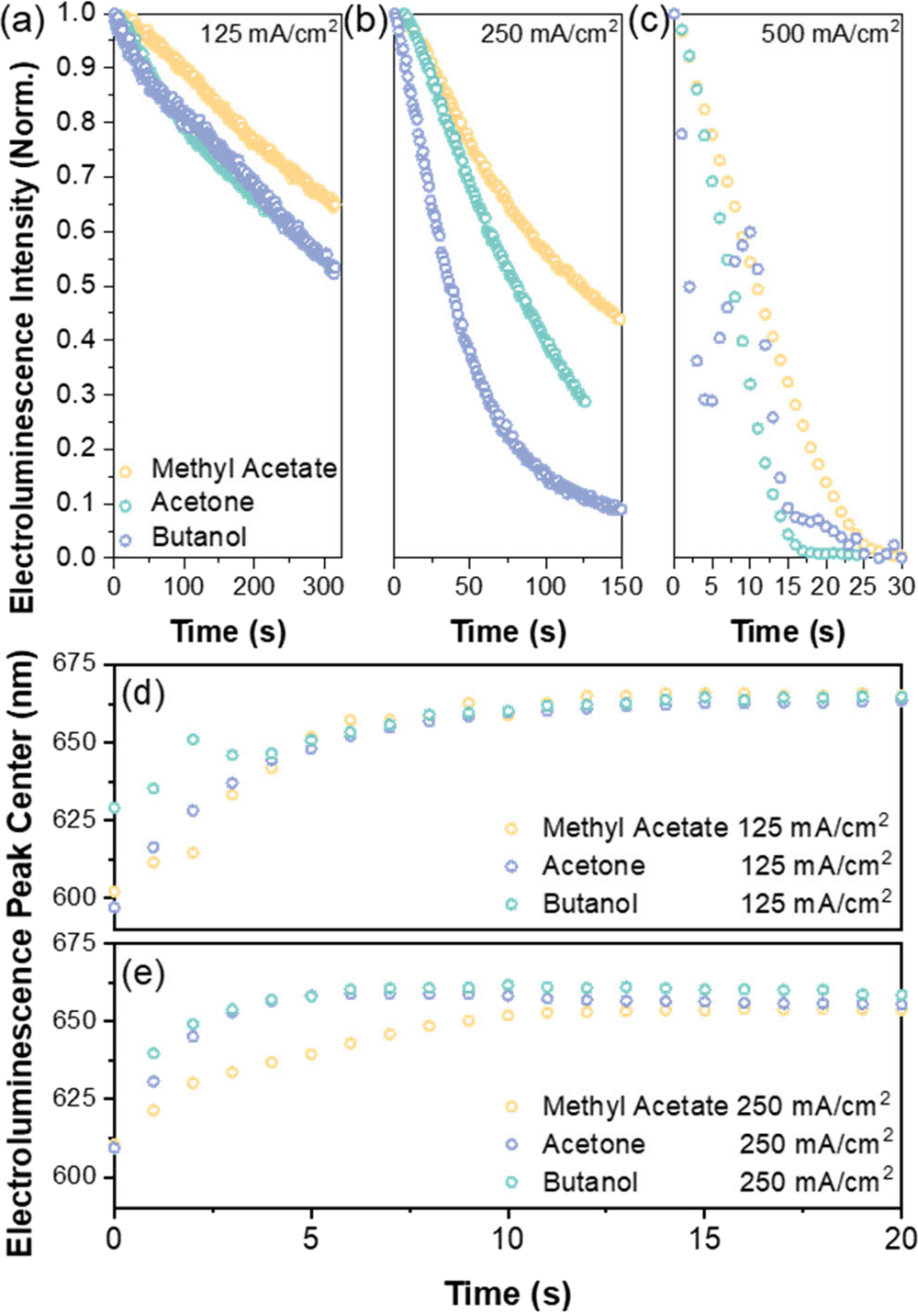
LED device stability. (a–c) Electroluminescence intensity over time at different current injection densities (125, 250, and 500 mA cm^–2^). (d, e) Change in the electroluminescence spectra peak center over time at 125 and 250 mA cm^–2^.

It is clear that devices made from NCs washed using low polarity antisolvents produced brighter EL that did not decay as quickly over time (Figures 6 and 7a–c). This is consistent with the PLQY and PL lifetime data reported in Figure 2. The expulsion of iodides during washing has influence on both device efficiency (EL intensity) and operational stability (EL decay speed). Furthermore, Figure 7d,e shows the effect on EL spectral stability at different injection levels. In both cases, the EL is gradually redshifted and stabilized at around 664 nm. The differences between the PL emission and the EL emission (redshift) are well observed and studied in many previous works.^50−52^ When an external voltage is applied to the NC films, the iodide ions tend to migrate and form a I-rich and Br-rich region on the film. The injected carriers will eventually radiatively recombine at places with lower bandgaps, i.e. the I-rich regions, causing the EL to shift to a lower energy position (Figure 7d,e). However, at 250 mA cm^–2^, methyl acetate-washed NC device clearly show a slower redshift compared to the NCs washed by higher-polarity solvents. We believe that the speed of ion migration is related to the initial halide vacancies present in the NC films. This is supported by our DFT calculations shown in Figure S15, SI. The climbing image nudged elastic band method was used to calculate the energy needed for iodide ions to move from one spot to adjacent vacancies at different initial halide vacancy concentration (Figure S15a, SI). The results show that for lower-initial vacancy concentrations, the ions need to overcome a 2.80 eV energy barrier to migrate. This situation corresponds to the case of NCs washed by lower polarity antisolvents, such as methyl acetate. In contrast, for higher-initial vacancy concentrations, the energy barrier reduced to 2.49 eV. This corresponds to the case for NCs washed by higher polarity antisolvents, such as acetone and butanol. Since the EL peak center for methyl acetate-washed NC device redshifts slower than for the two other solvents (Figure 7e), we believe that the NCs washed with lower-polarity antisolvents could induce a lower concentration of defects and hence improve device EL stability. An important direction for future work is to explore and develop antisolvents that induce lower surface damage to the NCs. A marginal reduction in the antisolvent relative polarity from methyl acetate (relative polarity = 0.253) by using ethyl acetate (relative polarity = 0.228) was insufficient to reduce the blueshift in PL after purification (Figure S19, SI), and alternative routes will need to be explored.

## Conclusions

In conclusion, this study aimed to unravel how the surface chemistry of all-inorganic mixed-halide (Br/I) perovskite NCs was influenced by the antisolvent during the purification process, which is a crucial step for preparing high-performance optoelectronic devices, such as light-emitting diodes and solar cells. Higher-polarity antisolvents led to increased selective etching of the surface halides (in this case iodide), resulting in increased sub-bandgap trap density and consequent reductions in PLQY and PL lifetimes. We put forward and examined two possible mechanisms to explain these effects. Based on NMR, XPS, and FTIR studies along with the DFT calculations, we believe the second hypothesis—amide evolution—to be the most likely. That is, we believe that the presence of polar antisolvents promotes the condensation reaction between the ligands to form amides that are no longer capable of binding to the surface of the NCs, which then leads to the removal of the surface halides that were bound to these ligands. Antisolvents with higher relative polarity tend to favor the shift in the OA-OAm equilibrium toward their acid/amine forms, thus promoting amide formation and increasing the removal of surface ligands and iodides. Minimizing these effects through the use of lower-polarity antisolvents led to brighter and more operationally-stable LEDs. By providing new fundamental insights into how antisolvents influence the surface chemistry of mixed-halide perovskite NCs, this work will guide the future design of NC synthesis routes not only for halide perovskites but also for other NC systems. In particular, this work shows that achieving purified NCs with higher PLQY that lead to more efficient and stable LEDs not only can be achieved by using lower-polarity antisolvents but could also be realized through the careful tuning of ligand–ligand and surface-ligand interactions to change the surface reactions that occur following the introduction of polar antisolvents.

## Supporting Information Available

Additional experimental details; TEM images; EDX; XPS; solution ^1^H-NMR; DFT calculations; TCSPC fittings (PDF)

## Notes

Raw data is available via https://doi.org/10.14469/hpc/10613.
