# Supplementary material for: Elucidating the Role of Antisolvents on the Surface Chemistry and Optoelectronic Properties of CsPbBrxI3-x Perovskite Nanocrystals
Source: J Am Chem Soc. 2022 Jun 27;144(27):12102–15. doi: 10.1021/jacs.2c02631 (PMC9284547; doi:10.1021/jacs.2c02631)
Supplement: Supplementary file 1 — ja2c02631_si_001.pdf [file ja2c02631_si_001.pdf]

Elucidating the Role of Antisolvents on the Surface Chemistry and Optoelectronic Properties of $CsPbBr_xI_{3-x}$ Perovskite Nanocrystals

Junzhi Ye [†], Zhenchao Li [‡], Dominik J. Kubicki [†, §], Yunwei Zhang [//, †], Linjie Dai [†], Clara Otero Martínez[#], Manuel A Reus [⊥], Rakesh Arul [†], Kavya Reddy Dudipala [‡‡], Zahra Andaji-Garmaroudi [†], Yi-Teng Huang [†], Zewei Li [†], Ziming Chen [‡], Peter Müller-Buschbaum [⊥, ∇], Hin-Lap Yip [‡, O], Samuel D. Stranks [†, ††], Clare P. Grey [§], Jeremy J Baumberg[†], Neil C. Greenham [†], Lakshminarayana Polavarapu [#], Akshay Rao [† *], Robert L. Z. Hoye [‡‡*]

[†] Cavendish Laboratory, University of Cambridge, JJ Thomson Ave, Cambridge CB3 0HE, United Kingdom

[‡] State Key Laboratory of Luminescent Materials and Devices, School of Materials Science and Engineering, South China University of Technology, 381 Wushan Road, Guangzhou 510640, P.R. China.

[§] Yusuf Hamied Department of Chemistry, University of Cambridge, Lensfield Road, Cambridge CB2 1EW, UK

[//] School of Physics, Sun Yat-sen University, 510275 Guangzhou, China

[#] CINBIO, Universidade de Vigo, Materials Chemistry and Physics Group, Department of Physical Chemistry, Campus Universitario As Lagoas, Marcosende, 36310 Vigo, Spain

[⊥] Lehrstuhl für Funktionelle Materialien, Physik-Department, Technische Universität München, James-Franck-Str. 1, 85748 Garching, Germany

[∇] Heinz Maier-Leibnitz Zentrum (MLZ), Technische Universität München, Lichtenbergstr. 1, 85748 Garching, Germany

[O] Department of Materials Science and Engineering, City University of Hong Kong, Tat Chee Avenue, Kowloon, Hong Kong.

[††] Department of Chemical Engineering & Biotechnology, University of Cambridge, Cambridge, CB3 0AS UK

[‡‡] Department of Materials, Imperial College London, Exhibition Road, London SW7 2AZ, UK

Email: ar525@cam.ac.uk (A.R.), r.hoye@imperial.ac.uk (R.L.Z.H.)

Keywords: Lead-halide perovskite, nanocrystals, solvent polarity, nuclear magnetic resonance, optoelectronic properties

Supplementary Experimental Details

Device Fabrication

ITO/glass substrates (Colorado Concept Coatings LLC) were cleaned by ultrasonication in acetone and isopropanol for 15 min each and dried in the oven. The substrates were subsequently O_2-plasma treated for 10 min at 250 W forward power (0 W reverse power), using a radio-frequency plasma source. For bipolar devices, poly(3,4- ethylene dioxythiophene) polystyrene sulfonate (PEDOT: PSS; Heraeus Clevios P Al.4083) was subsequently deposited at 3500 rpm for 30 s (1000 rpm s^{-1} acceleration), then annealed at 145 °C for 15 min. Poly(N,N'-bis(4-butyl phenyl)-N,N'-bis-phenyl benzidine) (poly-TPD; 1-Material) were dissolved in chlorobenzene (anhydrous 99.8%, Sigma Aldrich) with a concentration of 4 mg mL^{-1} . These polymer layers were spin-cast over the PEDOT: PSS at 2000 rpm for 30 s inside a nitrogen-filled glovebox and annealed at 120 °C for 10 min. The NCs were subsequently deposited at 1500 rpm for 30 s inside an N_2-filled glovebox. 35 nm of 2,2',2"-(1,3,5-benzinetriyl)-tris(1-phenyl-1-H-benzimidazole) (TPBi; Ossila) was thermally evaporated over the NCs under a base pressure of 1×10^{-6} mbar, followed by 1 nm LiF and 100 nm Al. The devices were measured in a glovebox.

Density Functional Theory Calculations

The first-principles calculations were carried out based on density functional theory (DFT) with the Perdew−Burke−Ernzerhof (PBE) exchange correlation functional as implemented in the Vienna ab initio Simulation Package (VASP). The all-electron projector-augmented wave (PAW) method was adopted. All of the DFT calculations were performed under periodic boundary conditions using the Vienna ab simulation package (VASP). The projector-augmented-wave (PAW) method with a plane-wave basis set was used. The plane-wave energy cutoff is set to 600 eV for $CsPbBr_{0.6}I_{2.4}$ unit cell. A Monkhorst−Pack Brillouin zone sampling grid with a resolution of $2\pi \times 0.03$ Å^{-1} is adopted to ensure that all the enthalpy calculations are well converged with an error less than 1 meV/atom. After the unit cell fully relaxed, a $2 \times 2 \times 2$ supercell was adopted to simulate the atomic position in a $CsPbBr_{0.6}I_{2.4}$ unit cell.

The energy barriers of halide ion migrations in these perovskites were studied by the nudged elastic band (NEB) method. To simulate the halide ion migration, a $2\times 2 \times 2$ supercell was used to calculate the vacancy formation energy and optimize the minimum energy pathway of halide ion migration. Five images (excluding initial and final states) are linearly interpolated between the initial and final states during the mitigation process.

Transmission Electron Microscopy

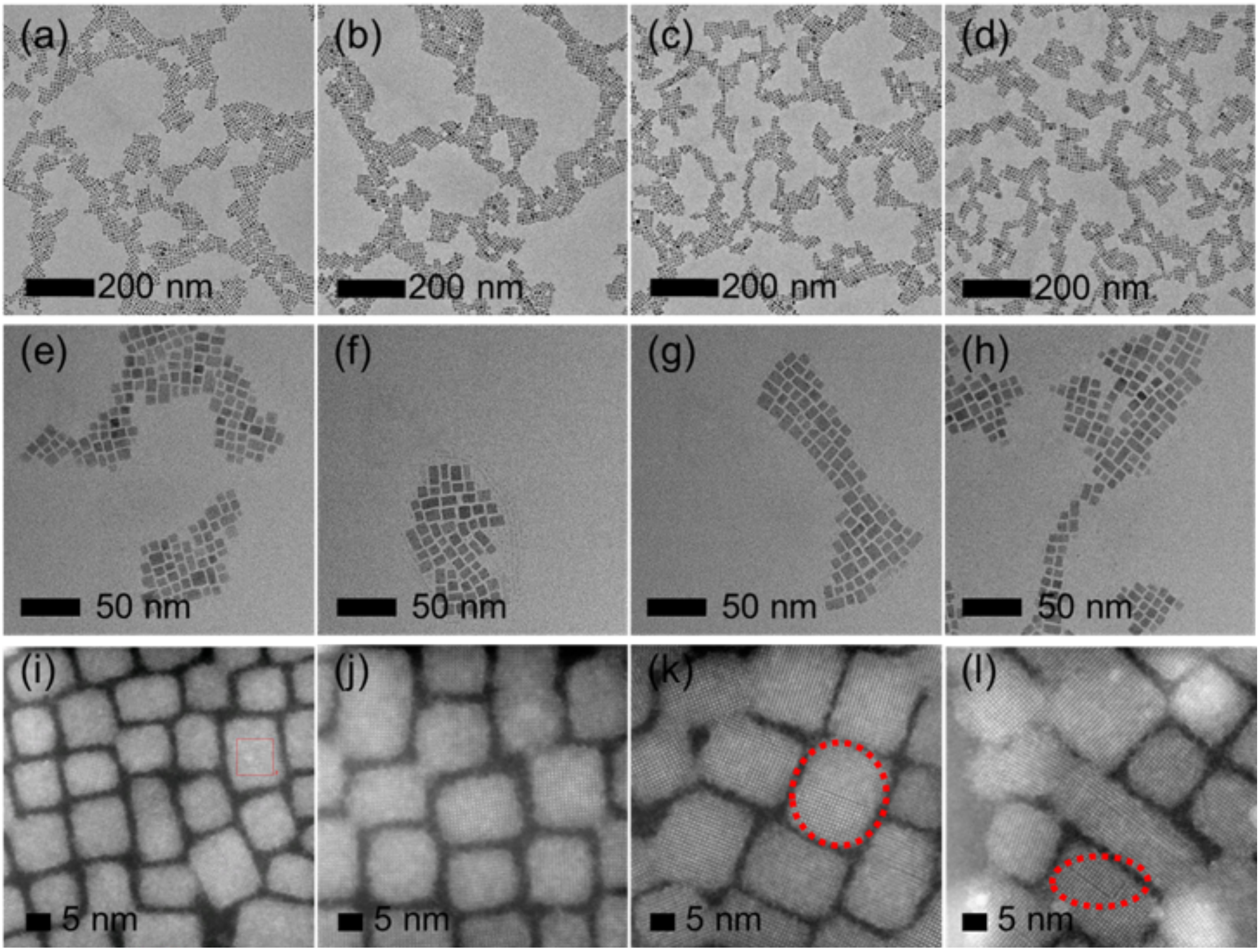

Figure S1 (a)-(h) TEM images. (i)-(l) STEM images. Image (a), (e) and (i) are original NCs. Image (b), (f) and (j) are NCs washed with methyl acetate. Image (c), (g) and (k) are NCs washed with acetone. Image (d), (h) and (l) are NCs washed with butanol.

Table S1. Composition study. TEM-EDX and XPS results for NC purified with and without antisolvents.

TEM EDX	Atomic Percentage (%)				Ratio (%)
Antisolvents	Cs	Pb	Br	I	I/(Br+I)
Original	14.44	10.54	13.39	61.63	82.15
Methyl Acetate	15.37	25.97	28.73	29.93	51.02
Acetone	18.03	23.39	37.22	21.37	36.47
Butanol	13.29	23.08	47.65	15.98	25.11
XPS	Atomic Percentage (%)				Ratio (%)
Antisolvents	Cs	Pb	Br	I	I/(Br+I)
Original	Covered by ligands (Atomic % cannot be resolved)				
Methyl Acetate	13.57	27.64	32.80	25.99	44.21
Acetone	12.91	29.36	47.52	10.21	17.69
Butanol	11.68	27.00	52.46	8.85	14.43

Table S2. XRD analysis to calculate lattice constant. X-ray wavelength is 1.5406 Å.

Antisolvents	Miller Indices			Bragg's Angle		d-spacing	Lattice Constant, a (Å)	Average a
	h	k	l	2θ	θ	$d_{hkl}=\lambda/(2\sin\theta)$	$a=d_{hkl}*\sqrt{h^2+k^2+l^2}$	(Å)
Butanol	1	0	0	14.6874	7.3437	6.0264	6.0264	6.0173
	2	0	0	29.7151	14.8575	3.0041	6.0082	
Acetone	1	0	0	14.6705	7.3353	6.0333	6.0333	6.0282
	2	0	0	29.6394	14.8197	3.0116	6.0232	
Methyl Acetate	1	0	0	14.4988	7.2494	6.1044	6.1044	6.0838
	2	0	0	29.4391	14.7195	3.0316	6.0633	
Original	1	0	0	14.4415	7.2208	6.1284	6.1284	6.1074
	2	0	0	29.3246	14.6623	3.0432	6.0864	

Optical Characterization

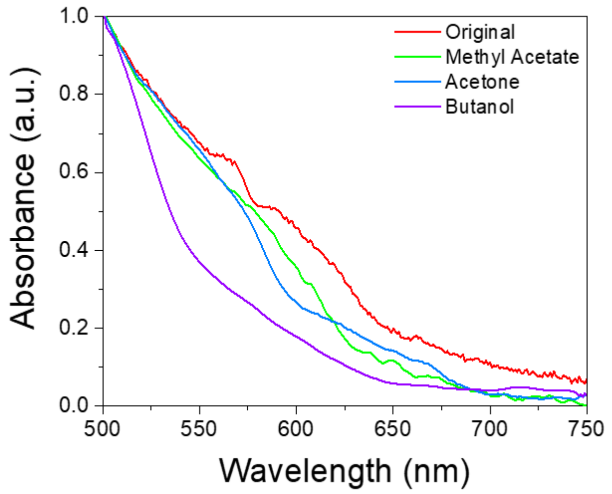

Figure S2 UV-Vis absorption spectra of washed NC films prepared by spin-coating.

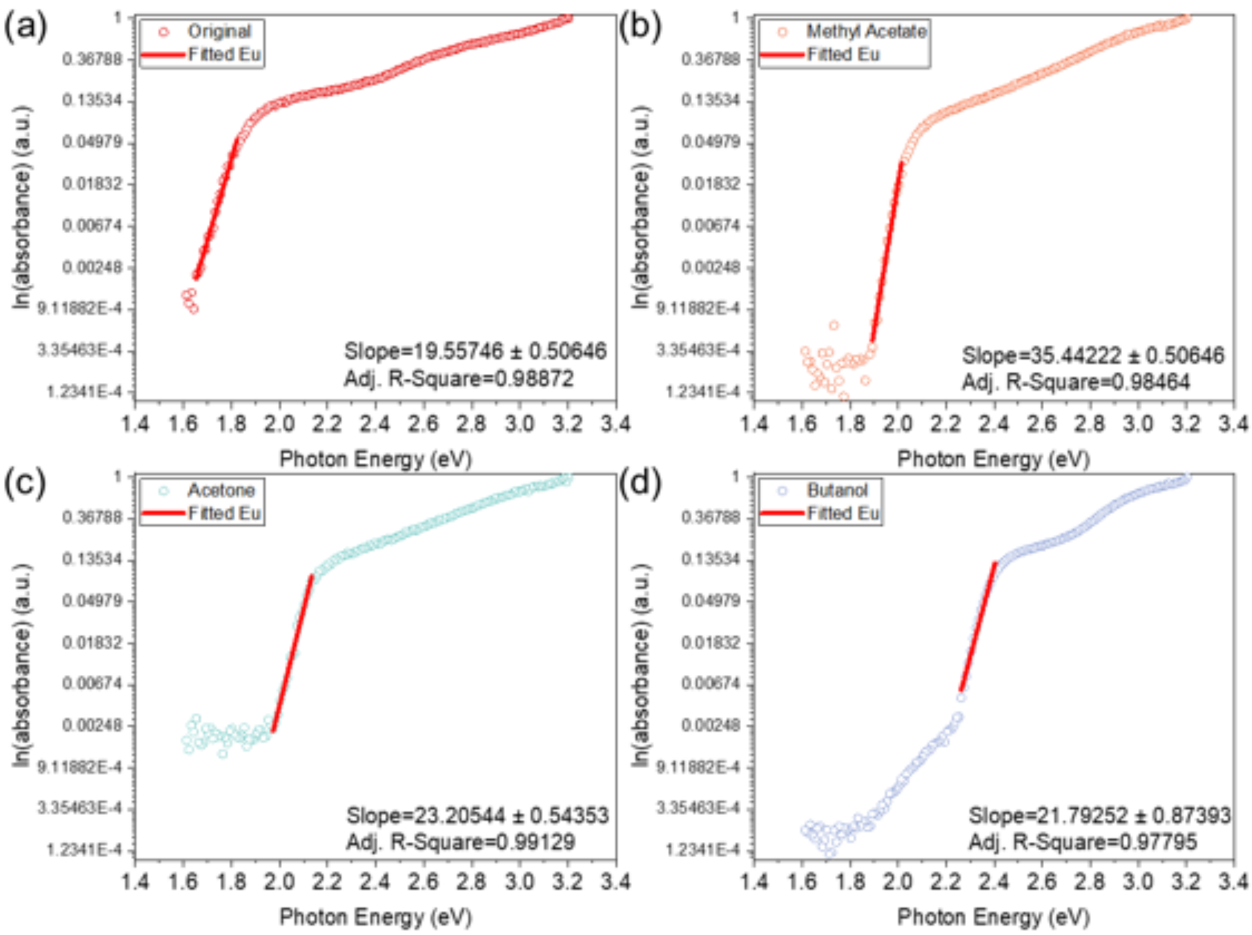

Figure S3. Fitted PDS data to extract Urbach Energy. (a) Original NC films, (b) methyl acetate washed NCs, (c) acetone washed NCs, (d) butanol washed NCs.

Urbach Energy Fitting

The Urbach energy of the NC films can be calculated by equation (S1) and (S2).

$$\alpha = \alpha_0 + e^{\frac{E}{E_u}} \qquad \text{(S1)}$$

$$E_u = \frac{\log(\alpha - \alpha_0)}{E} = \frac{1}{\text{slope}} \qquad \text{(S2)}$$

where α is the absorption coefficient, E is the energy of the light absorbed, and E_u the Urbach energy.

Table S3. Fitted Urbach energy of NC thin films.

Samples	Slope	E_u (meV) (1/slope)
Original	19.6	51.0
Methyl Acetate	35.4	28.2
Acetone	23.2	43.1
Butanol	21.8	45.9

We note that in fitting the model given in equation S1 to the PDS data in Figure S3, we are assuming that there is Urbach absorption in the energy range considered. However, this region is convoluted with the excitonic peak superimposed over the rise in the absorption continuum. The Urbach energies obtained and shown in Table S3 are therefore approximate values. Regardless, we can see from Figure S3 that the NCs treated with methyl acetate had steeper absorption onsets than NCs purified with acetone or butanol, which is either due to lower Urbach energies or narrower excitonic peaks, with can both arise due to reduced disorder in the NCs.

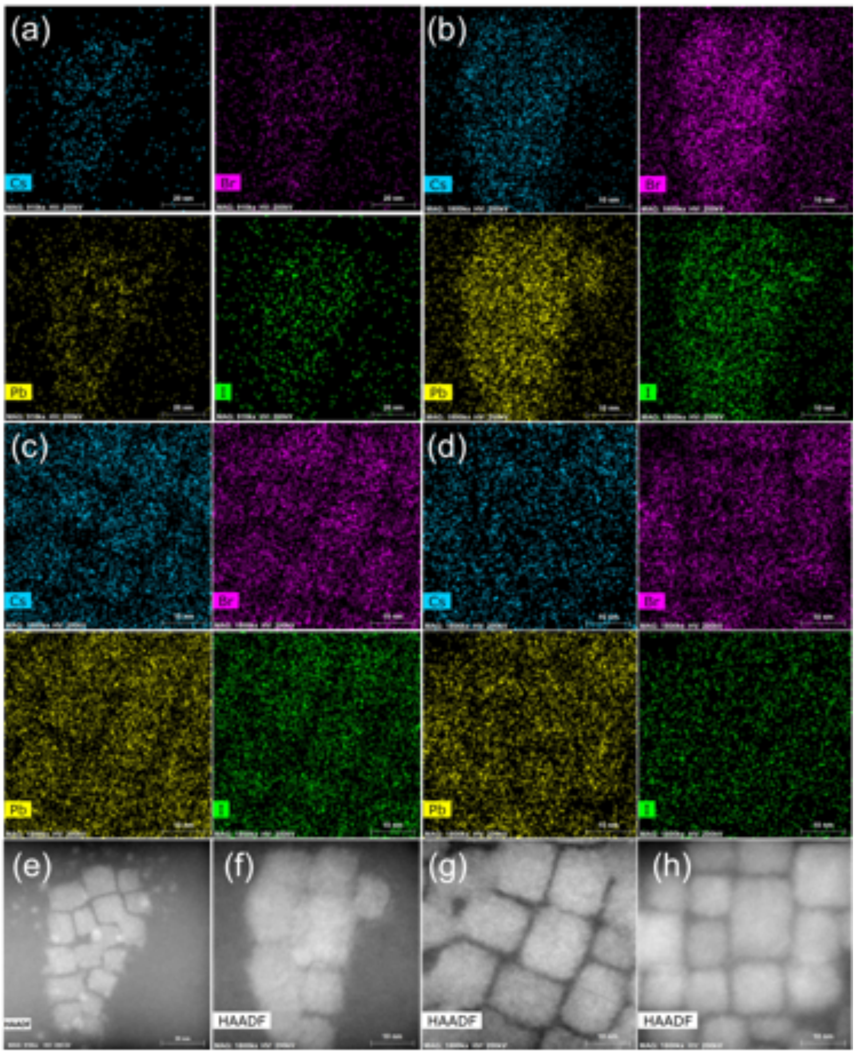

Figure S4 TEM EDX elemental mapping of a) NCs purified without antisolvent, and NCs purified with b) methyl acetate, c) acetone, d) butanol, along with (e)-(h) their corresponding HADDF TEM images.

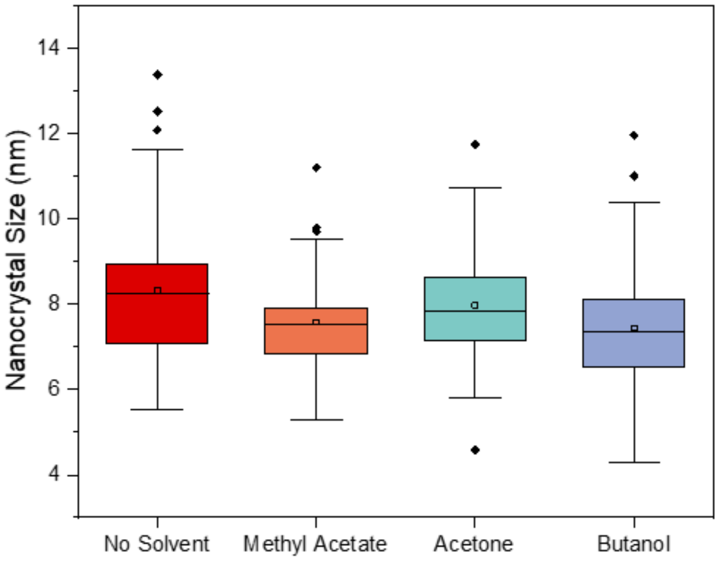

Figure S5 Box plots of the nanocrystal size distribution.

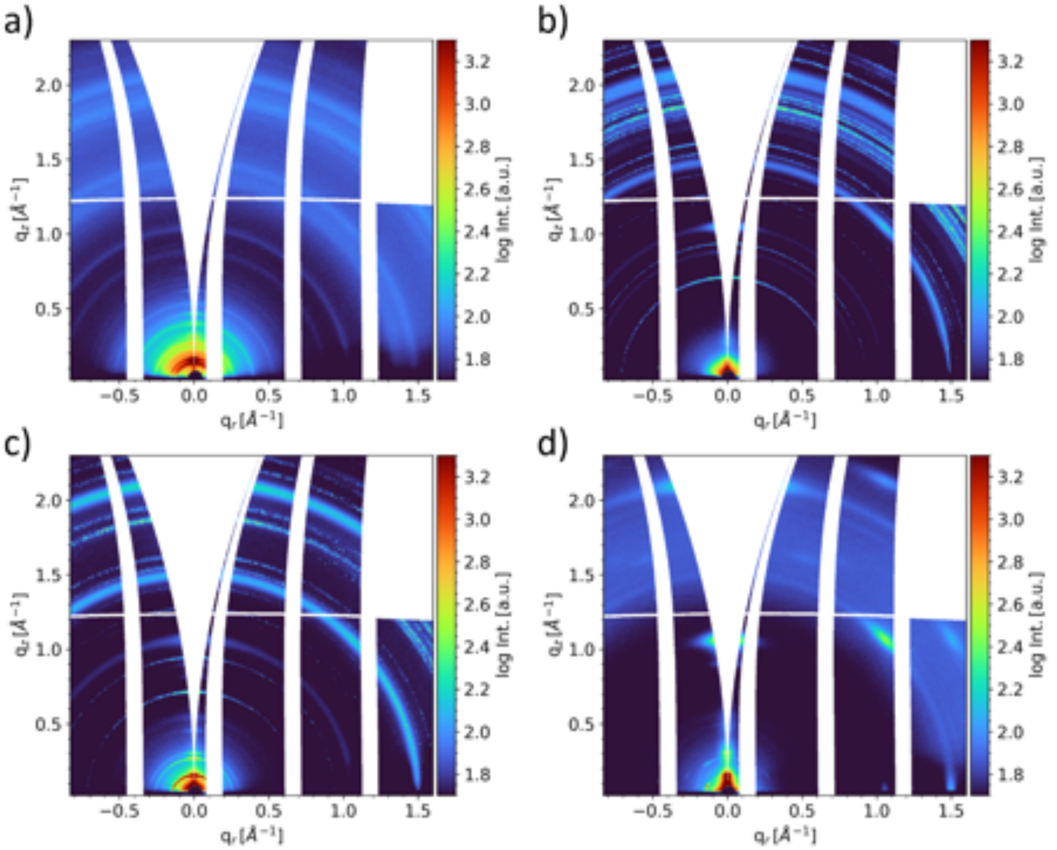

Figure S6 2D GIWAXS data of NCs a) not washed and washed with b) methyl acetate, c) butanol and d) acetone.

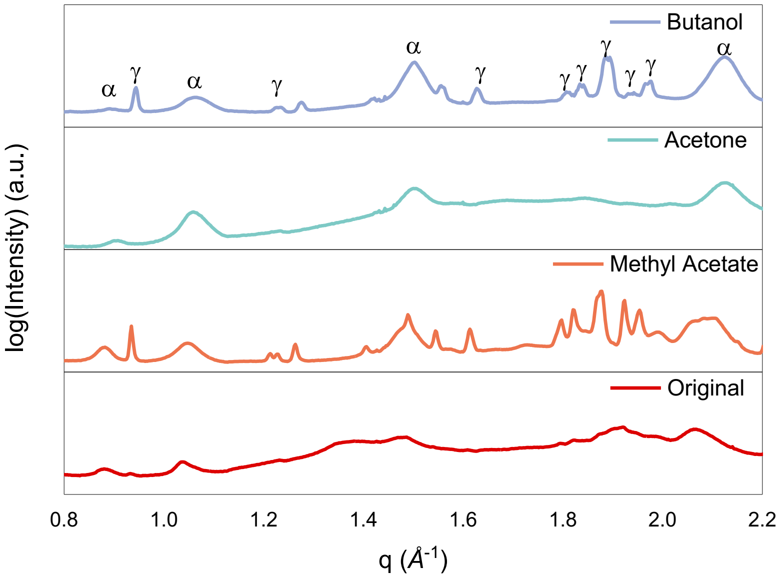

Figure S7 GIWAXS line cut plot. Perovskite phases are labelled as cubic phase (α) and orthorhombic phase (γ). In the GIWAXS measurements, we observed that there was a mixture of cubic phase (α) and orthorhombic phase (γ) in the NC films. The orthorhombic phase may come from film degradation during the GIWAXS measurement.[1-2]

TCSPC Fitting

Table S4. Fitted Fluence dependent TCSPC from 0.1 nJ cm^{-2} pulse^{-1} to 0.39 nJ cm^{-2} pulse^{-1}

Sample 0.39 nJ/cm2	$t_{average}$	t_1	A_1	t_2	A_2	t_3	A_3
Original	16.40	1.72E+00	2.30E-01	7.81E+00	4.97E-01	2.41E+01	2.12E-01
Methyl Acetate	9.03	7.97E-01	3.66E-01	4.52E+00	4.73E-01	1.43E+01	1.61E-01
Acetone	11.17	8.44E-01	1.25E-01	5.48E+00	5.05E-01	1.46E+01	3.32E-01
Butanol	9.07	8.31E-01	4.24E-01	4.29E+00	3.74E-01	1.49E+01	1.21E-01

Sample 0.29 nJ/cm2	$t_{average}$	t_1	A_1	t_2	A_2	t_3	A_3
Original	16.79	1.37E+00	2.22E-01	7.09E+00	5.27E-01	2.38E+01	2.44E-01
Methyl Acetate	9.55	7.19E-01	4.87E-01	4.60E+00	5.74E-01	1.44E+01	2.29E-01
Acetone	10.51	1.02E+00	1.32E-01	5.26E+00	5.16E-01	1.38E+01	3.39E-01
Butanol	6.82	4.08E-01	8.89E-01	3.03E+00	5.16E-01	1.06E+01	2.04E-01

Sample 0.19 nJ/cm2	$t_{average}$	t_1	A_1	t_2	A_2	t_3	A_3
Original	18.87	2.35E+01	2.20E-01	6.76E+00	2.93E-01	1.35E+00	1.01E-02
Methyl Acetate	10.99	9.32E-01	3.37E-01	5.58E+00	5.87E-01	1.61E+01	2.57E-01
Acetone	10.44	7.06E-01	2.34E-01	4.97E+00	5.33E-01	1.37E+01	3.56E-01
Butanol	4.45	3.92E+00	4.56E-01	6.73E-01	3.48E+00	1.27E+01	9.36E-02

Sample 0.10 nJ/cm2	$t_{average}$	t_1	A_1	t_2	A_2	t_3	A_3
Original	21.05	7.11E+00	1.17E-01	2.41E+01	1.60E-01	1.61E+00	2.92E-04
Methyl Acetate	14.58	2.13E+00	2.03E-01	8.88E+00	5.95E-01	2.24E+01	2.01E-01
Acetone	10.25	1.14E+00	1.78E-01	5.91E+00	5.62E-01	1.48E+01	2.44E-01
Butanol	3.67	2.95E+00	4.26E-01	4.47E-01	5.71E+00	1.04E+01	1.30E-01

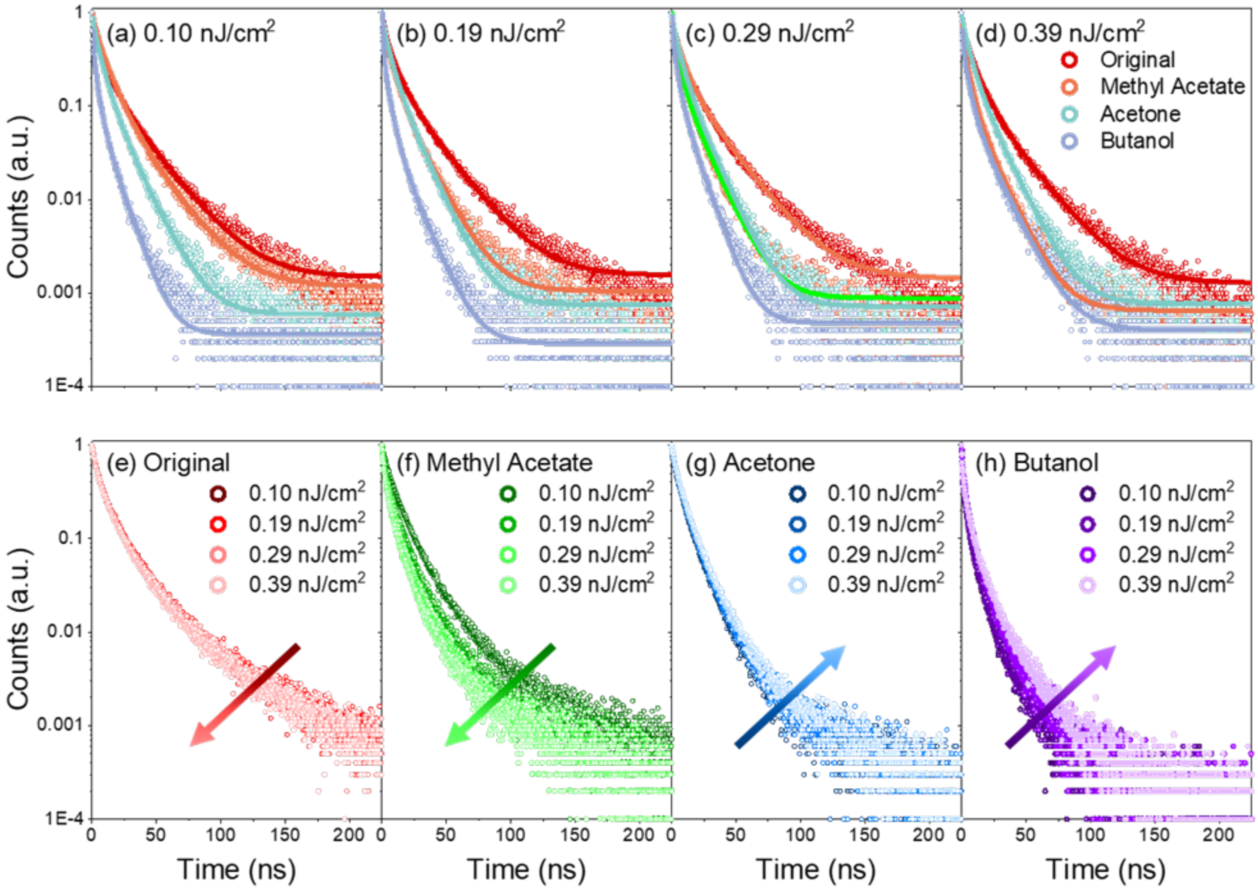

Figure S8 TCSPC. (a)-(d) Fitted TCSPC at different fluence. (e)-(h) Fluence Dependent TCSPC.

The fitting is based on the following equation (S3 and S4):

$$y = y_0 + A_1 e^{-(x-x_0)/t_1} + A_2 e^{-(x-x_0)/t_2} + A_3 e^{-(x-x_0)/t_3} \qquad \text{(S3)}$$

$$\tau_{average} = \frac{A_1 \times {t_1}^2 + A_2 \times {t_2}^2 + A_3 \times {t_3}^2}{A_1 \times t_1 + A_2 \times t_2 + A_3 \times t_3} \qquad \text{(S4)}$$

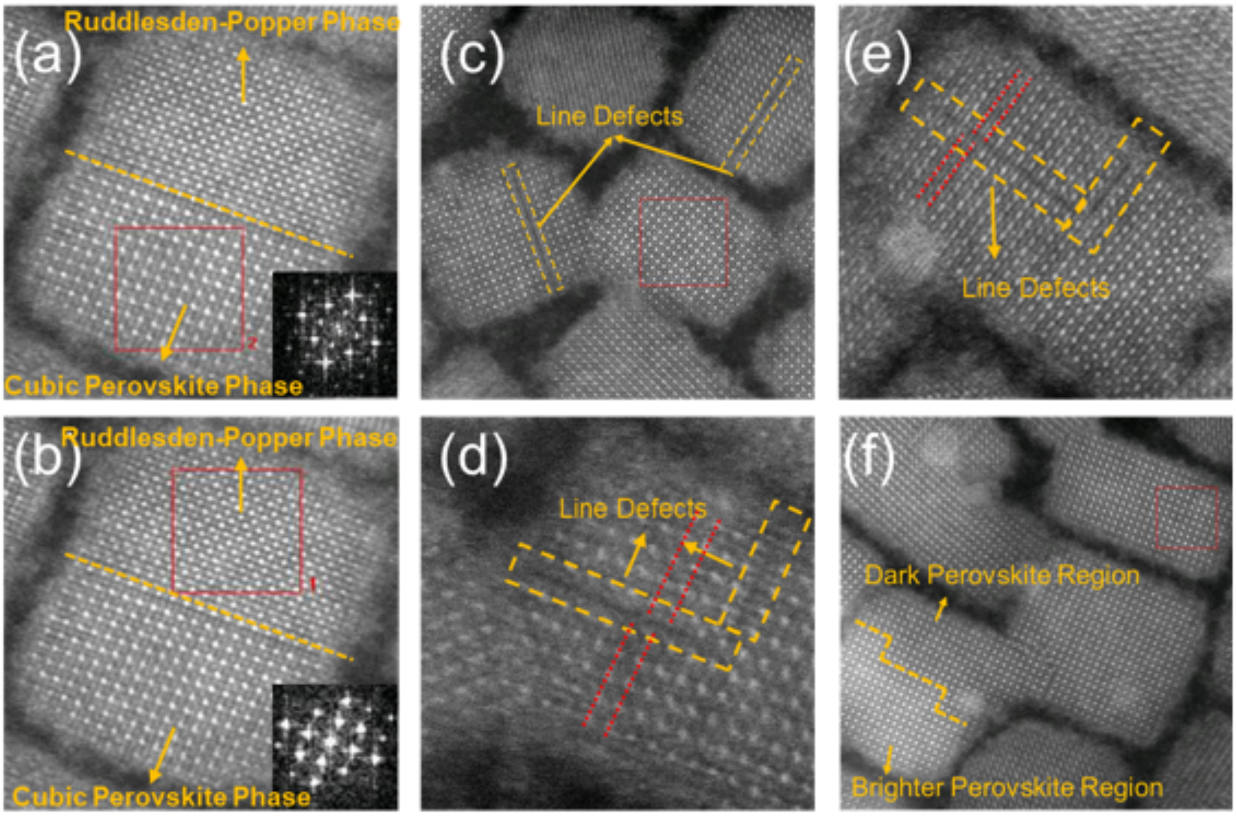

Figure S9 STEM mode of High-Angle Annular Dark-Field imaging (HAADF) to reveal the structural defects in purified NCs. (a,b) HADDF images of acetone-washed NCs, with the fast Fourier transform (FFT) of the area shown in the red square shown inset. These data show that after washing with acetone, there is a possibility of introducing Ruddlesden-Popper phase into the cubic phase perovskite NCs (top-half of the NC imaged). (c), (d) and (e) focus on the line defects introduced after washing the NCs with (c) methyl acetate, (d) acetone, and (e) butanol. (f) STEM image of washed NCs which show regions with different brightness on the same NC, which could indicate a change in the NC surface termination, or a change in the thickness after washing.

Density Functional Theory

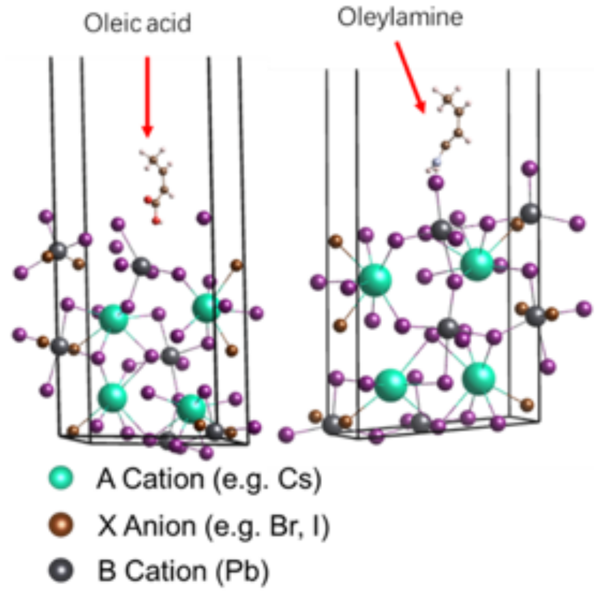

Figure S10 Density functional theory calculations. The NC is bound to oleylammonium ligand through hydrogen bonding from the amine group to the halide. The oleic acid or oleate ions are also attached closely to NC surface by balancing the surface charge of the NCs, mostly Pb^{2+} states.

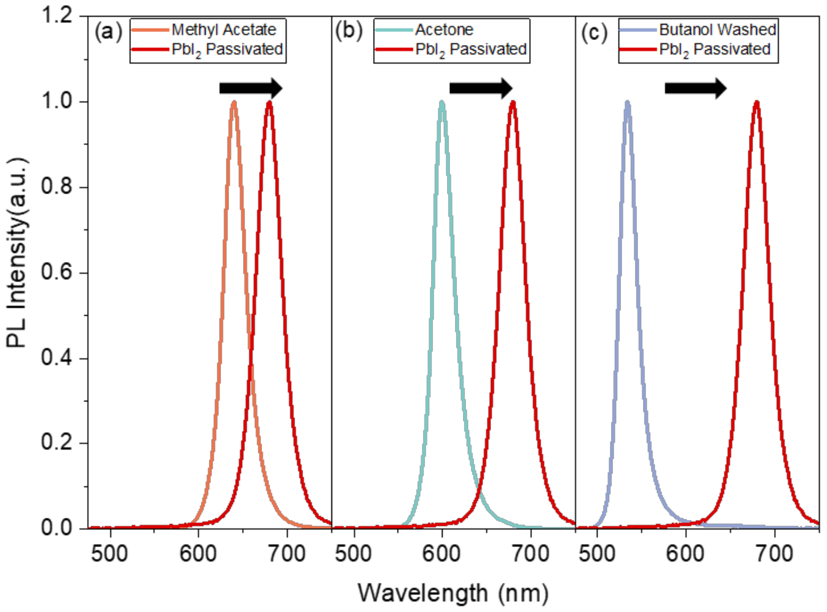

Figure S11 PL spectra for NCs re-passivate with PbI_2 precursors. (a) Methyl acetate, (b) acetone and (c) Butanol.

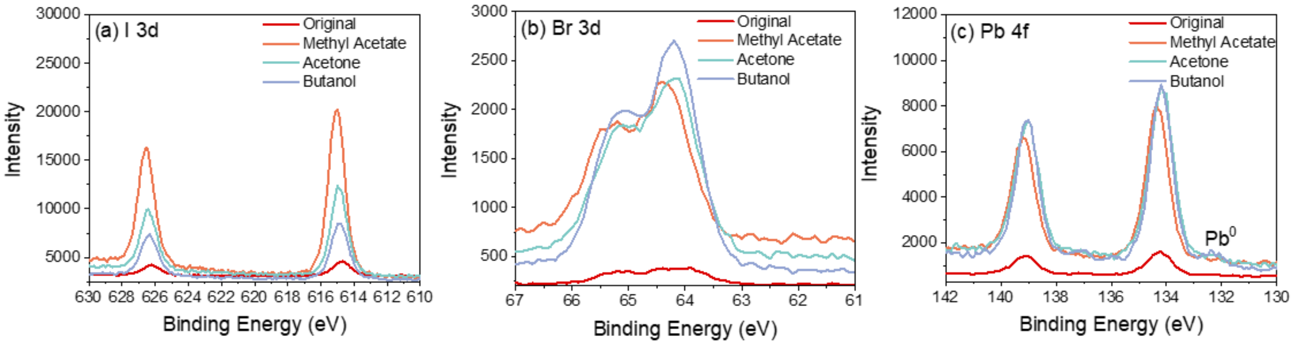

Figure S12 XPS spectra of (a) I 3d, (b) Br 3d and (c) Pb 4f core level.

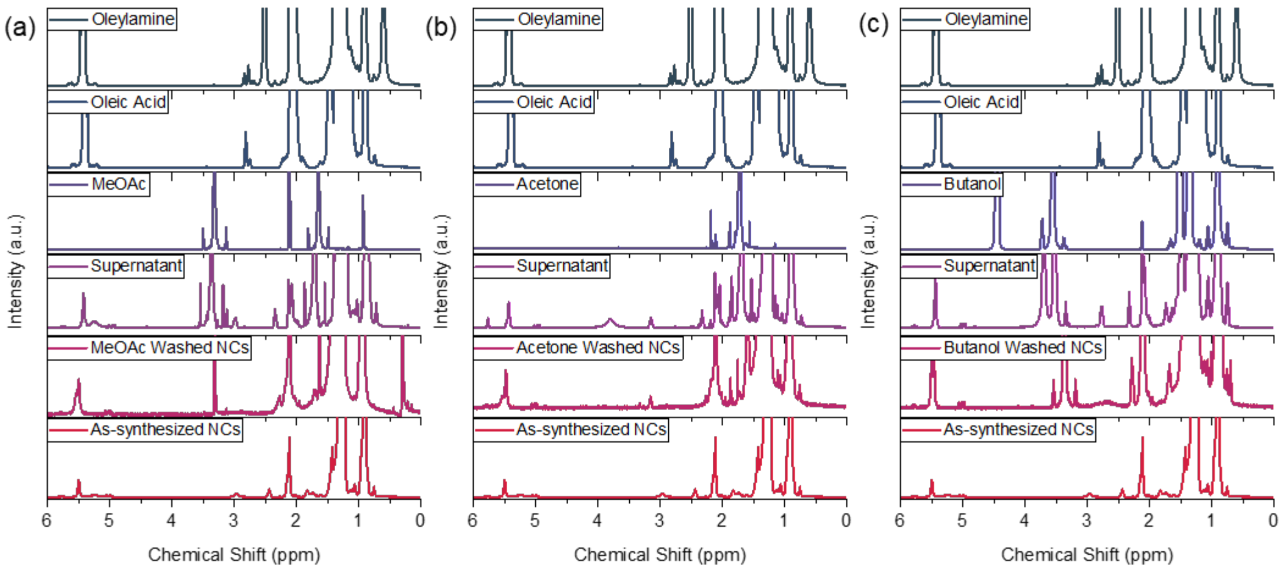

Figure S13 ^{1}H-NMR spectra of antisolvents, ligands, original NCs, and washed NCs with their supernatant solutions. (a) Methyl acetate series. (b) Acetone series (c) Butanol series.

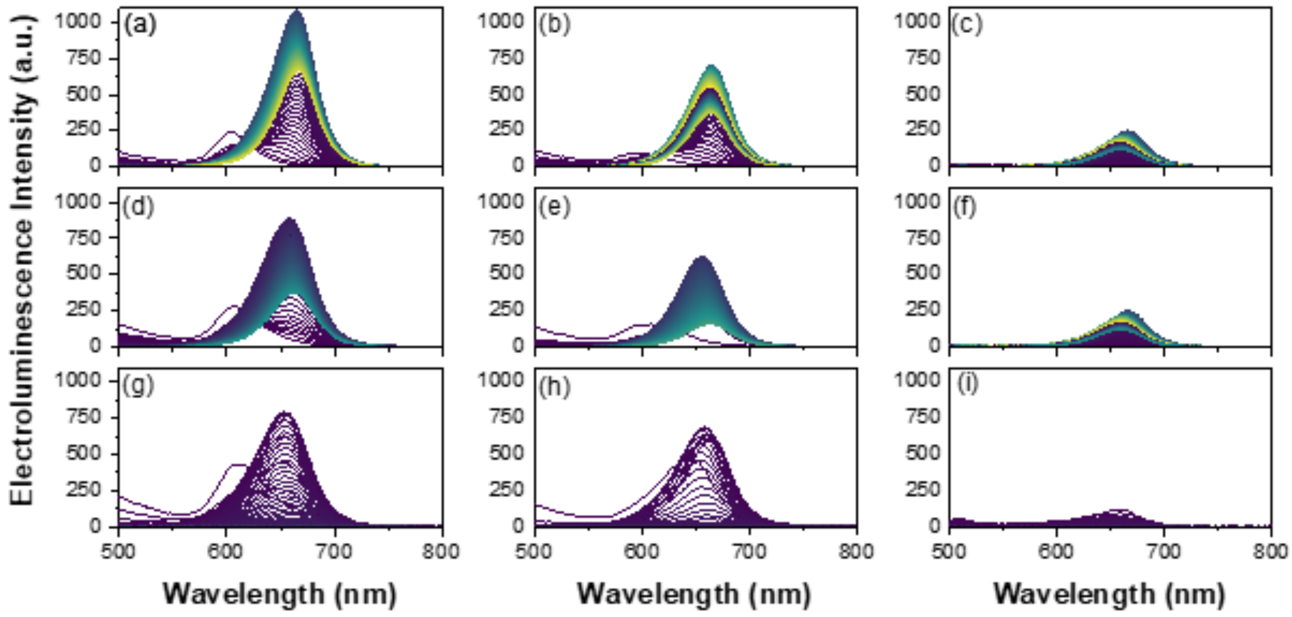

Figure S14 Electroluminescence Spectra of perovskite LEDs. (a),(d) and (g) are NCs washed by methyl acetate.

(b), (e) and (h) are NCs washed by acetone and (c), (f) and (i) are NCs washed by butanol. (a)-(c) are measured at 125 mA cm^{-2}. (d)-(f) are measured at 250 mA cm^{-2}. (g)-(i) are measured at 500 mA cm^{-2}.

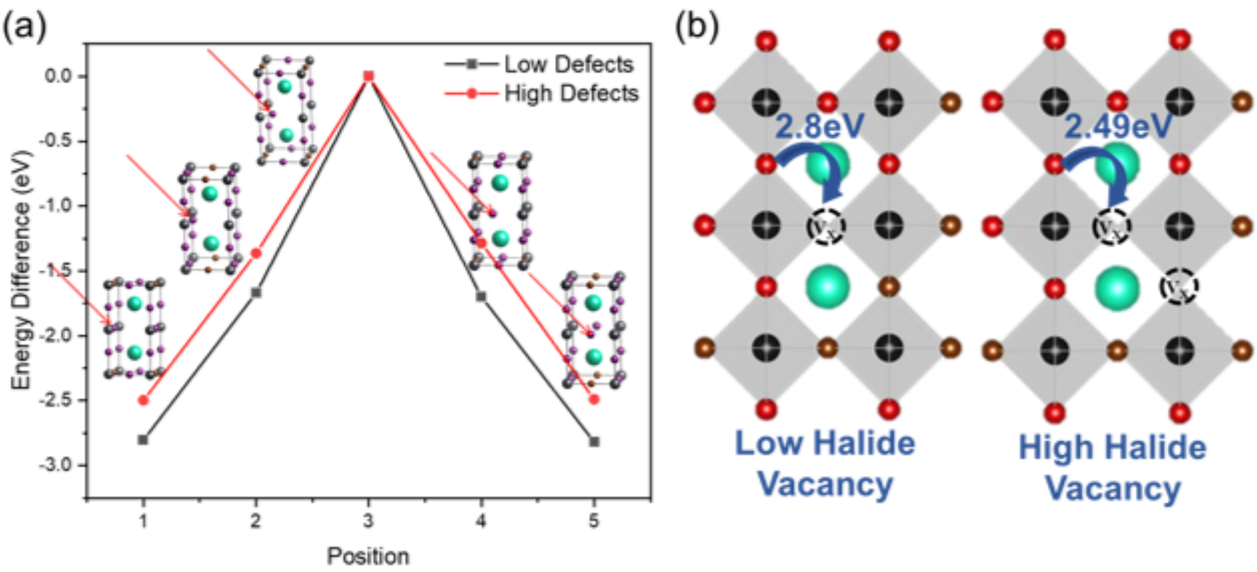

Figure S15 DFT calculation to explain the halide phase segregation during device operation. (a) Climbing Image Nudged Elastic Band method to calculate the energy needed for iodide to move from one spot to adjacent vacancies at different initial halide vacancy concentration.(b) Schematic diagram to show the energy needed for the halide to migrate in perovskite lattices with different initial halide vacancy concentrations.

Table S5. PL shift of washed $CsPbBr_xI_{3-x}$ NCs prepared through Br halide exchange from $CsPbI_3$ NCs

Washing Solvent	Solvent Polarity	PL Peak (nm)
**Hexane**	**0.009**	**605**
**Methyl acetate**	**0.253**	**593**
**Acetone**	**0.355**	**588**
**Butanol**	**0.552**	**565**

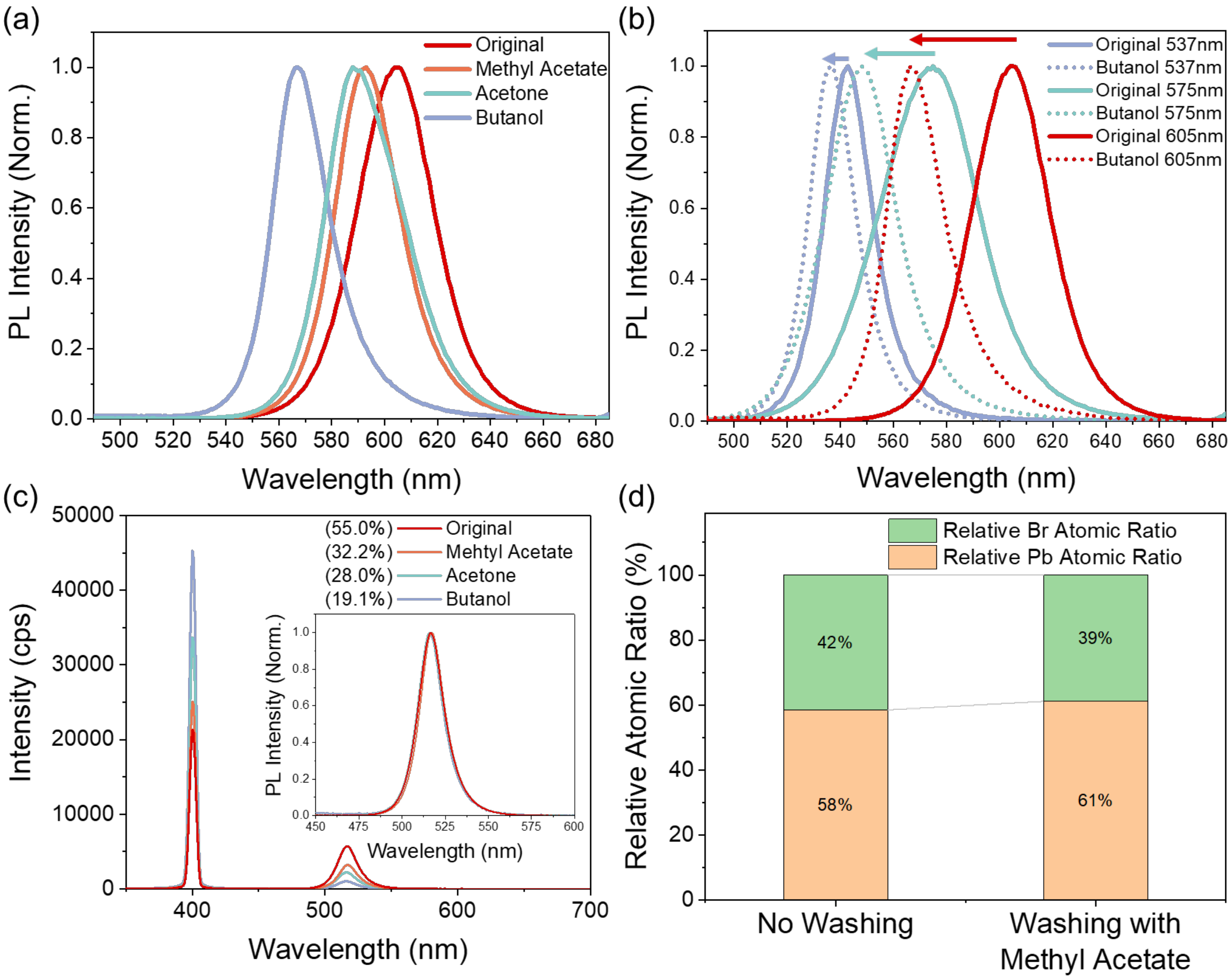

Figure S16 Washing studying on $CsPbBr_xI_{3-x}$ NCs prepared by Br exchange starting from $CsPbI_3$ and pure $CsPbBr_3$ NCs. (a) PL spectra of $CsPbBr_xI_{3-x}$ NCs prepared by Br exchange starting from $CsPbI_3$ and purified with methyl acetate, acetone and butanol antisolvents (b) PL spectra of $CsPbBr_xI_{3-x}$ NCs with different Br/I ratio prepared by Br exchange starting from $CsPbI_3$ and purified with butanol antisolvents (c) PL spectra and PLQYs of $CsPbBr_3$ NCs purified with methyl acetate, acetone and butanol antisolvents, compared to purification without antisolvents ("Original"). The PLQYs are shown in the brackets in the labels. (d) Surface Br and Pb atomic ratio of the original NCs and methyl acetate washed NCs obtained based on XPS results.

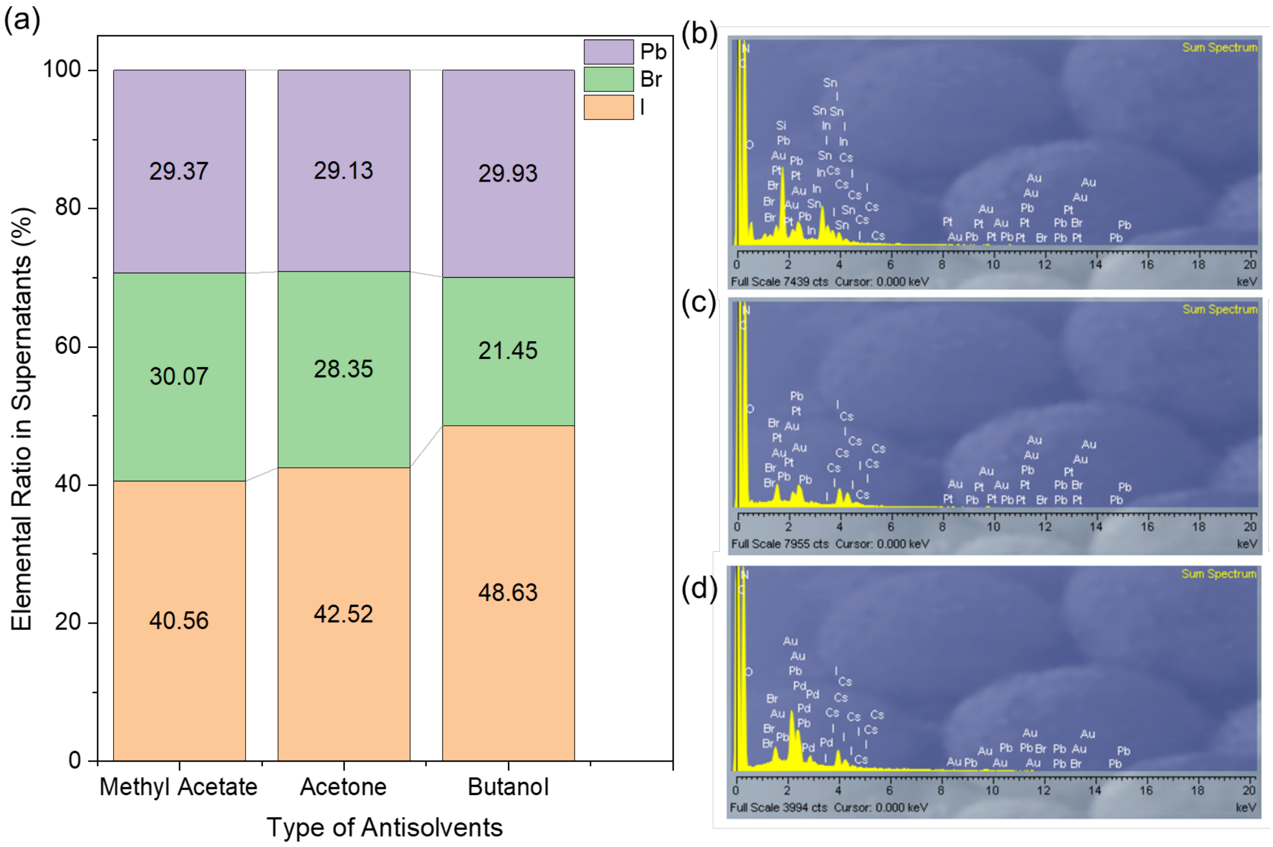

Figure S17 SEM-EDX of the drop cast supernatants obtained from colloidal NCs washed by different antisolvents. The SEM used is LEO 1530 VP instrument and the EDX detector is from Oxford Instruments. (a) Atomic ratio of Pb, I and Br on drop-casted supernatants prepared by washing the NCs with butanol, acetone and methyl acetate. Screen shot of the SEM-EDX of supernatants obtained following NC purification using (b) methyl acetate, (c) acetone, and (d) and butanol. The samples were prepared by dropping 100 µL of supernatant solution onto ITO/glass substrates.

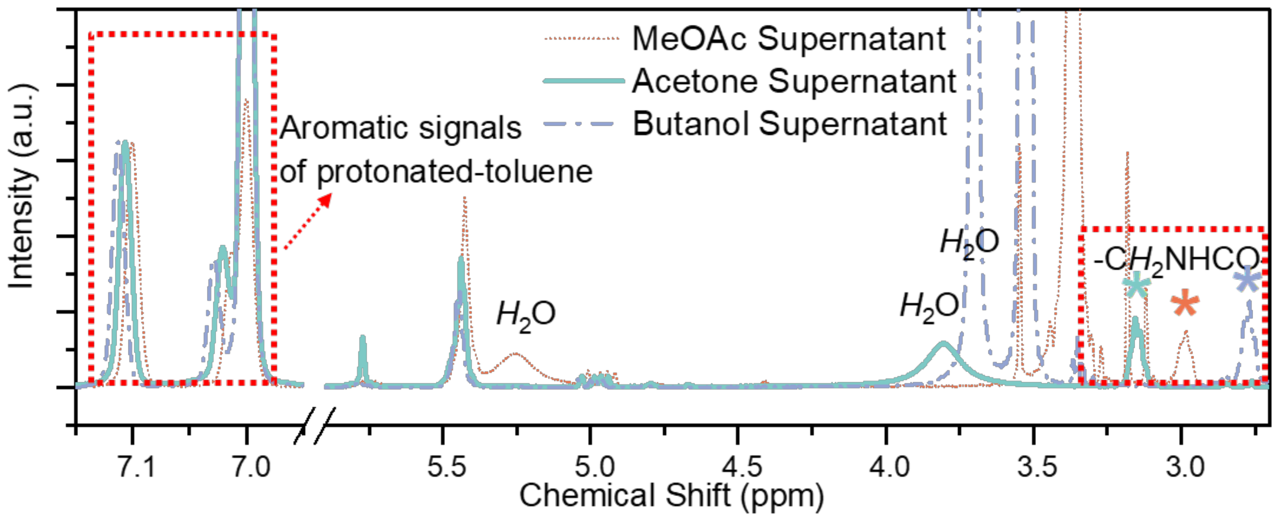

Figure S18 ^{1}H-NMR spectrum of the supernatants (normalized to the aromatic signal of residual protonated toluene in the d-6 toluene). The H-species characterized in the NMR peak is italicized.

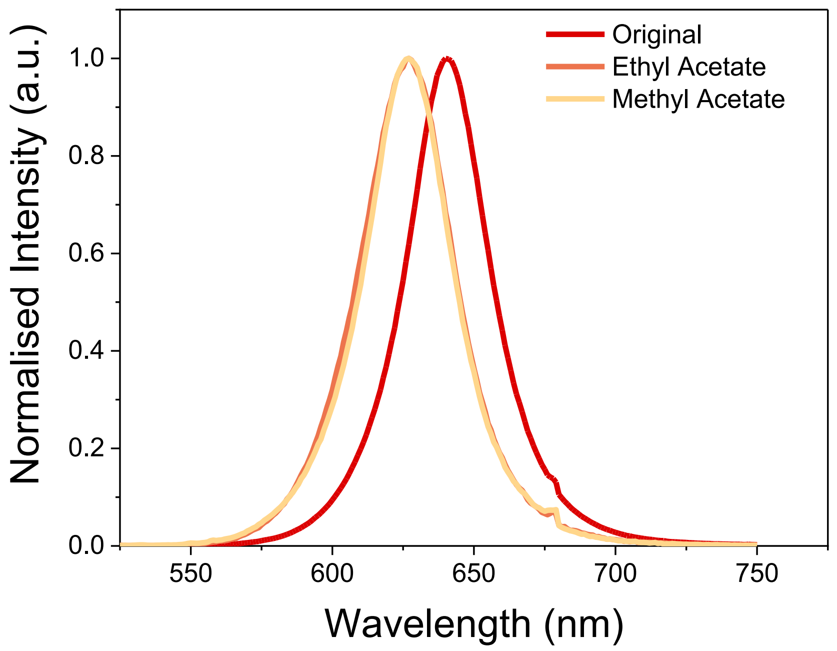

Figure S19 Photoluminescence spectra of the original NCs and NCs washed by methyl acetate and ethyl acetate.

References

1. Dey, A.; Ye, J.; De, A.; Debroye, E.; Ha, S. K.; Bladt, E.; Kshirsagar, A. S.; Wang, Z.; Yin, J.; Wang, Y.; Quan, L. N.; Yan, F.; Gao, M.; Li, X.; Shamsi, J.; Debnath, T.; Cao, M.; Scheel, M. A.; Kumar, S.; Steele, J. A.; Gerhard, M.; Chouhan, L.; Xu, K.; Wu, X.-g.; Li, Y.; Zhang, Y.; Dutta, A.; Han, C.; Vincon, I.; Rogach, A. L.; Nag, A.; Samanta, A.; Korgel, B. A.; Shih, C.-J.; Gamelin, D. R.; Son, D. H.; Zeng, H.; Zhong, H.; Sun, H.; Demir, H. V.; Scheblykin, I. G.; Mora-Seró, I.; Stolarczyk, J. K.; Zhang, J. Z.; Feldmann, J.; Hofkens, J.; Luther, J. M.; Pérez-Prieto, J.; Li, L.; Manna, L.; Bodnarchuk, M. I.; Kovalenko, M. V.; Roeffaers, M. B. J.; Pradhan, N.; Mohammed, O. F.; Bakr, O. M.; Yang, P.; Müller-Buschbaum, P.; Kamat, P. V.; Bao, Q.; Zhang, Q.; Krahne, R.; Galian, R. E.; Stranks, S. D.; Bals, S.; Biju, V.; Tisdale, W. A.; Yan, Y.; Hoye, R. L. Z.; Polavarapu, L., State of the Art and Prospects for Halide Perovskite Nanocrystals. *ACS Nano* **2021,** *15* (7), 10775–10981.
2. Thomas, C. J.; Zhang, Y.; Guillaussier, A.; Bdeir, K.; Aly, O. F.; Kim, H. G.; Noh, J.; Reimnitz, L. C.; Li, J.; Deepak, F. L.; Smilgies, D.-M.; Milliron, D. J.; Korgel, B. A., Thermal Stability of the Black Perovskite Phase in Cesium Lead Iodide Nanocrystals Under Humid Conditions. *Chemistry of Materials* **2019,** *31* (23), 9750-9758.
